# Hecate/Grip2a Acts to Reorganize the Cytoskeleton in the Symmetry-Breaking Event of Embryonic Axis Induction

**DOI:** 10.1371/journal.pgen.1004422

**Published:** 2014-06-26

**Authors:** Xiaoyan Ge, Danielle Grotjahn, Elaine Welch, Jamie Lyman-Gingerich, Christiana Holguin, Eva Dimitrova, Elliot W. Abrams, Tripti Gupta, Florence L. Marlow, Taijiro Yabe, Anna Adler, Mary C. Mullins, Francisco Pelegri

**Affiliations:** 1 Laboratory of Genetics, University of Wisconsin – Madison, Madison, Wisconsin, United States of America; 2 Department of Cell and Developmental Biology, Perelman School of Medicine at the University of Pennsylvania, Philadelphia, Pennsylvania, United States of America; Stanford University School of Medicine, United States of America

## Abstract

Maternal homozygosity for three independent mutant *hecate* alleles results in embryos with reduced expression of dorsal organizer genes and defects in the formation of dorsoanterior structures. A positional cloning approach identified all *hecate* mutations as stop codons affecting the same gene, revealing that *hecate* encodes the Glutamate receptor interacting protein 2a (Grip2a), a protein containing multiple PDZ domains known to interact with membrane-associated factors including components of the Wnt signaling pathway. We find that *grip2a* mRNA is localized to the vegetal pole of the oocyte and early embryo, and that during egg activation this mRNA shifts to an off-center vegetal position corresponding to the previously proposed teleost cortical rotation. *hecate* mutants show defects in the alignment and bundling of microtubules at the vegetal cortex, which result in defects in the asymmetric movement of *wnt8a* mRNA as well as anchoring of the kinesin-associated cargo adaptor Syntabulin. We also find that, although short-range shifts in vegetal signals are affected in *hecate* mutant embryos, these mutants exhibit normal long-range, animally directed translocation of cortically injected dorsal beads that occurs in lateral regions of the yolk cortex. Furthermore, we show that such animally-directed movement along the lateral cortex is not restricted to a single arc corresponding to the prospective dorsal region, but occur in multiple meridional arcs even in opposite regions of the embryo. Together, our results reveal a role for Grip2a function in the reorganization and bundling of microtubules at the vegetal cortex to mediate a symmetry-breaking short-range shift corresponding to the teleost cortical rotation. The slight asymmetry achieved by this directed process is subsequently amplified by a general cortical animally-directed transport mechanism that is neither dependent on *hecate* function nor restricted to the prospective dorsal axis.

## Author Summary

One of the earliest and most crucial events in animal development is the establishment of the embryonic dorsal axis. In amphibians and fish, this event depends on the transport of so-called “dorsal determinants” from one region of the egg, at the pole opposite from the site where the oocyte nucleus lies, towards the site of axis induction. There, the dorsal determinant activates the Wnt signaling pathway, which in turn triggers dorsal gene expression. Dorsal determinant transport is mediated by the reorganization of a cellular network composed of microtubules. We determine that *hecate*, a zebrafish gene active during egg formation that is essential for embryonic axis induction, is required for an early step in this microtubule reorganization. We find that *hecate* corresponds to *glutamate receptor interacting protein 2a*, which participates in other animal systems in Wnt-based pathways. We also show that the microtubule reorganization dependent on *hecate* results in a subtle symmetry-breaking event that subsequently becomes amplified by a more general transport process independent of *hecate* function. Our data reveal new links between *glutamate receptor interacting protein 2a*, Wnt signaling and axis induction, and highlights basic mechanisms by which small changes early in development translate into global changes in the embryo.

## Introduction

Dorsoventral axis formation is a fundamental process in early vertebrate embryogenesis. In many vertebrates such as amphibians and teleosts, evidence indicates that maternally-supplied dorsal determinants trigger the formation of the future dorsal organizer. Embryological manipulations have indicated that the dorsal determinants are initially localized to the vegetal pole and subsequently translocate animally to the prospective dorsal side in a microtubule-dependent process in both amphibians (reviewed in [1]) and teleosts [2]–[4]. In amphibians, translocation of the signal from the vegetal pole to the dorsal side is initiated by cortical rotation, the microtubule-dependent movement of the egg cortex with respect to its core that is triggered by fertilization and is implemented by transport along microtubule tracks (reviewed in [1]).

Although not readily apparent in the zebrafish embryo, detailed analysis has indicated the existence of processes that share similarities with the amphibian cortical rotation. Early studies showed that fluorescent polystyrene beads injected at the vegetal pole were transported animally along microtubule-based cortical tracks in a microtubule dependent manner [2], and that this movement had temporal dynamics and functional requirements similar to that of the movement of putative dorsal determinants as defined by embryological manipulations [3], [4]. Subsequent work showed that maternal factors such as Syntabulin and Wnt8a, involved in axis induction, localized to the vegetal pole of the egg and upon fertilization and egg activation shifted animally towards the presumed prospective dorsal region of the embryo [5]–[7]. Further studies of microtubule rearrangements in live embryos confirmed that the tracks of bundled microtubules that form at the zebrafish vegetal pole upon egg activation become aligned in the direction of the future dorsal side of the embryo, and showed bulk cortical particle movement analogous to a cortical rotation [8].

The movement of the dorsal determinant results in the activation of the Wnt/βcatenin signaling pathway and the activation of β-catenin-dependent targets [1]. This well-known pathway is characterized by the activation of Frizzled receptors by the Wnt ligand, and an intracellular cascade involving the activation of Dishevelled and the downregulation of a β-catenin degradation complex that includes GSK3, Axin and Adenomatous polyposis coli (APC), leading to the accumulation of β-catenin in the nucleus [9]. Nuclear β-catenin in turn interacts with transcription factors of the Tcf family to activate transcription of target genes. Wnt/βcatenin pathway components and/or nuclear accumulation of β-catenin have been shown to be involved in embryonic axis determination in diverse deuterostomes such as fish, amphibians, mammals and amphioxus, as well as in lineages as basal as echinoderms, Cnidarians and planaria (reviewed in [10]) implying that the pathway was recruited for axis determination very early in animal evolution.

Although the involvement of Wnt/βcatenin activation across species is well documented, the identity of the molecules that activate the pathway in the early embryo, often referred to as dorsal determinants, remains unknown in most cases. In *Xenopus*, *wnt11* mRNA is first located at the vegetal pole and becomes enriched at the future dorsal side after fertilization, and depletion of *wnt11* mRNA results in embryos defective in dorsal axis induction [11]. Thus, Wnt11, together with ubiquitously present Wnt5 [12], [13] has been proposed to be the dorsal determinant in this amphibian species. Studies in the zebrafish exclude a function for Wnt11 or Wnt 5 in axis induction but suggest a role for Wnt8a in this process [7]. Maternal zebrafish *wnt8a* mRNA is localized during oogenesis to the vegetal pole of the oocyte and, upon fertilization, *wnt8a* mRNA experiences a shift from its original location at the vegetal pole to an off-center region thought to correspond to the dorsal side [7]. These studies suggest that, while Wnt/β-catenin pathway activation may be highly conserved in axis induction across the animal kingdom, maternally-based mechanisms that lead to the activation of the pathway vary.

Efforts from several laboratories have used forward genetics approaches to identify maternal factors essential for various aspects of early embryonic development in the zebrafish [5], [14]–[18]. Several reports have documented maternal-effect mutations affecting zebrafish dorsal axis induction [5], [15], [17], [18]. Mutations in three maternal-effect genes, *ichabod*, *tokkaebi* and *hecate*, cause specific ventralized phenotypes, consistent with a role for these genes in axis induction. Overexpression of Wnt signaling pathway components in *ichabod* mutant embryos indicate that this mutation acts within the Wnt/β-catenin signaling pathway at a level downstream of the β-catenin degradation complex [15]. These and other results show that *ichabod* corresponds to a β-catenin-2 gene expressed maternally and involved in axis induction [19].

Similar overexpression analysis has shown that *tokkaebi* and *hecate*, in contrast to *ichabod*, act upstream of the β-catenin-degradation machinery [5], [20]. Specifically, overexpression of components that activate the pathway at multiple points can rescue the *hecate* and *tokkaebi* mutant phenotypes. Rescue by an exogenous Wnt ligand suggests that the Wnt/β-catenin pathway is intact in *tokkaebi* and *hecate* mutant embryos, and suggests that, rather than being required for an integral component of Wnt/β-catenin signaling, these genes are required to regulate an endogenous signal that activates this pathway. Consistent with such a proposed role, positional cloning reveals that *tokkaebi* corresponds to the *syntabulin* (*sybu*) gene, which codes for a linker of the kinesin I motor protein involved in cargo transport along microtubules [6]. Both *sybu* mRNA and protein are localized to the vegetal pole of the egg, and Sybu protein exhibits a slight off-center shift upon egg activation [6]. These data suggest a role for Sybu in the microtubule-dependent transport of vegetally localized dorsal determinants.

Here, we present the molecular characterization of the zebrafish maternal-effect gene *hecate (hec)*. Maternal homozygosity for three independent mutant *hec* alleles results in embryos with reduced expression of dorsal organizer genes and defects in the formation of dorsoanterior structures ([20]; this report). Positional cloning reveals that *hec* encodes the Glutamate receptor interacting protein 2a (Grip2a). We find that *grip2a* mRNA, like *wnt8a* mRNA and Sybu protein, is localized to the vegetal pole of the oocyte and early embryo, where during egg activation it is shifted off-center corresponding to the previously proposed teleost cortical rotation [8]. The Drosophila Grip homologue has recently been shown to potentiate Wnt signaling at the neuromuscular junction by interacting with the Frizzled receptor on the cytoplasmic side of endocytosing membrane vesicles [21], [22], suggesting a potential mechanism of action for zebrafish Grip2a in axis induction at the level of Frizzled receptor regulation. Unexpectedly, however, we find that *hec* mutants show defects in the alignment and bundling of microtubules at the vegetal cortex, which result in corresponding defects in the asymmetric movement of *wnt8a* mRNA and are sufficient to explain the observed axis induction defects.

The short-range shift in vegetally localized factors such as *grip2a* mRNA also led us to re-examine the functional significance of the previously observed animally-directed cortical transport on axis induction. We find that, although short-range shifts in vegetal signals are affected in *hec* mutant embryos, these mutants do not exhibit a defect in the long-range, animally directed translocation of cortically injected dorsal beads that occurs in lateral regions of the yolk cortex. Furthermore, we show that, contrary to our expectations, such movements are not restricted to a single arc corresponding to the prospective dorsal region, but occur in multiple meridional arcs even in opposite regions of the embryo. Together, our results propose a role for *hec* function in the reorganization and bundling of microtubules at the vegetal cortex to mediate a symmetry-breaking event likely corresponding to the teleost cortical rotation. This asymmetry is subsequently amplified by a cortical animally-directed transport mechanism that is neither dependent on *hec* function nor restricted to the prospective dorsal axis.

## Results

### Maternal-effect mutations in *hecate* affect dorsoanterior development

**Figure 1:**
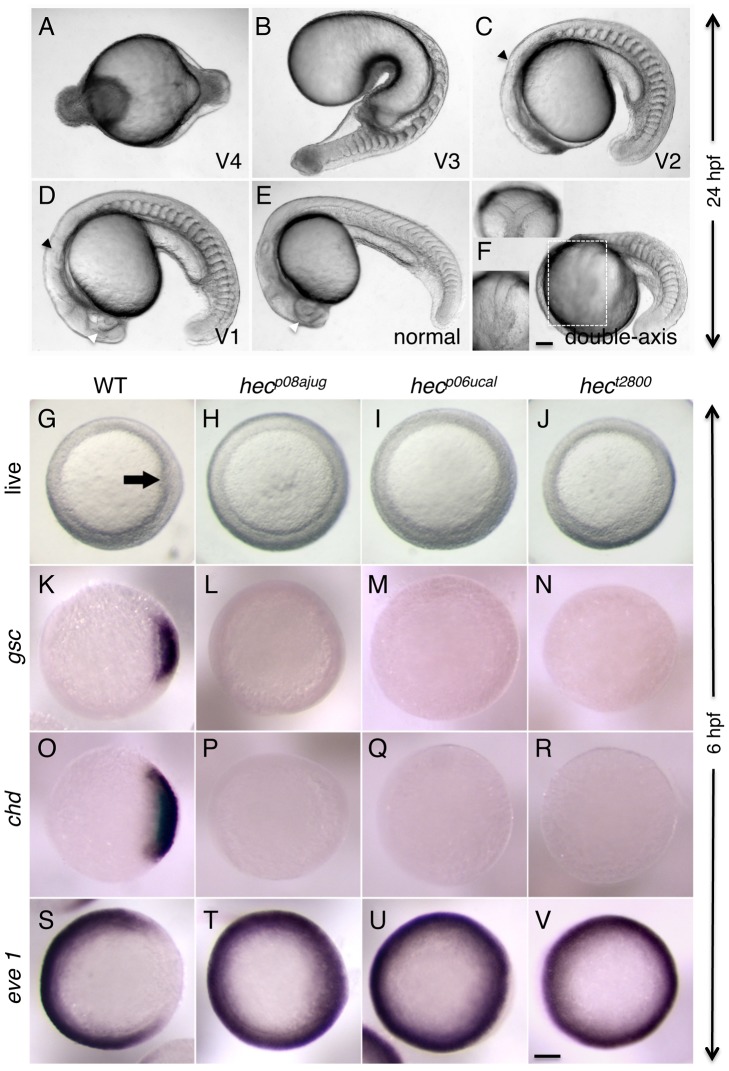
Axis induction defects in embryos from mothers homozygous for three different *hecate* mutant alleles. A–F) Side views (except as indicated in (F)) of live embryos at 24 hpf showing a range of phenotypes from most severe (A) to normal (E) and double-axis embryos (F). A) Class V4 embryo exhibiting complete radial symmetry and lacking all anterior (e.g. head structures) or dorsalmost (e.g. notochord) structures. B) Class V3 embryo exhibiting a rudimentary axis but lacking all anterior and dorsalmost structures. C) Class V2 embryo lacking anteriormost structures such as the eyes, but showing formation of more posterior head structures such as the otic vesicles (black arrowhead, also indicated in D). D) Class V1 embryo with a relatively complete axis but reduced anterior structures, such as the eyes (white arrowhead, also labeled in E), and lacking a properly formed notochord. E) Normal embryo derived from *hec* mutant females indistinguishable from wild-type. Note somites in (E) are chevron-shaped, while they are blocky in (B–D) indicative of defects in notochord formation, or encircling the entire embryo in (A). F) A double-axis embryo found in *hec* mutant clutches exhibiting weak expressivity. Insert in the lower left shows the left axis indicated by the dashed rectangle, out of focus in the main image. Insert in the upper left panel shows a dorsal view, showing the bifurcated axis. Note the lack of defined anterior structures in both axes, as well as the lack of a notochord along the trunk, also reflected by block-shaped somites in this region. Images in A–F are side views, except for upper left insert in (F). G–I) Animal views of live wild-type embryos (G) and embryos from females homozygous for each of the three *hec* mutant alleles. The dorsal thickening or shield (arrow) is absent in mutant embryos. K–V) In situ hybridization analysis to detect expression of dorsally-expressed genes (*gsc*, *chd*) and ventrally-expressed genes (*eve 1*). The expression domains of *gsc* and *cho* is reduced in *hec* mutant embryos, while the expression domain of *eve* is expanded in these embryos. (K–V) are animal view of embryos, dorsal to the right when identifiable, at the shield stage (6 hpf). Magnification bars in (F) and (V) correspond to 100 µm for panels sets (A–F) and (G–V), respectively. Dorsal view insert in panel (F, upper left) has been reduced to 75% size.

Embryos from mothers homozygous for the mutant *hec* allele, for simplicity referred to here as *hec* mutant embryos, display a range of ventralized phenotypes [17], [20], [23] (Figure 1A–E; Table 1). In the most severe cases, 24-hour mutant embryos exhibit a severe radial ventralization and lack all dorsoanterior-derived structures (V4 class, according to criteria in [24]; Figure 1A). More moderate phenotypes can also be observed, such as embryos that lack all anterior structures as well as the notochord (a dorsally-derived structure), and display an expansion of posterior somites (V3 class; Figure 1B). More weakly ventralized embryos are also observed that lack the anterior-most head structures and the notochord and exhibit expanded posterior somites (V2 class; Figure 1C), and embryos with reduced eyes and some notochord defects (class V1; Figure 1D). In many clutches, a fraction of embryos from mutant mothers are indistinguishable from wild-type embryos (Figure 1E). Such variability in phenotypes can be observed in maternal-effect mutants (some examples can be found in [17], [25], [26]), in particular those exhibiting axis induction defects [5], [15], [27], possibly by inherent variation in the maternal composition of individual eggs coupled to gene or pathway redundancy (see also Discussion). In mutant clutches with a weak expression of the phenotype, a small and variable fraction of embryos exhibits axis duplication phenotypes (Figure 1F) instead of axis formation defects (see Discussion).

**Table 1:** Phenotypic strength of *hecate* alleles.

Genotype	V4^1^ (%)	V3^2^ (%)	V2^3^ (%)	V1^4^ (%)	Double axis^5^ (%)	Normal (%)	n
WT^6^	0.2	0.2	0.0	0.1	0.0	99.5	1259
*hec^p06ucal^*	76.3	7.7	6.5	8.7	0.0	0.9	1812
*hec^t2800^*	46.7	13.1	9.5	15.0	0.3	15.4	655
*hec^p08ajug^*	10.9	18.9	15.4	28.8	3.1	22.8	1516

Embryos from females homozygous for *hec* mutant alleles were scored at 24 hpf for ventralization phenotypes according to [24]. Results were pooled from clutches from 15 different females for each allele.

1) Severe radial ventralization and lack of all dorsoanterior structures.

2) Absence of anterior structures, including posterior head structures such as the otoliths, lack of notochord, and expanded posterior somites.

3) Absence of anterior head structures including the eyes, lack of notochord, and expanded posterior somites.

4) Reduced anterior head structures such as the eyes but otherwise normal.

5) In weak mutant clutches, a small fraction of embryos exhibit a duplicated axes, each of which often exhibits deletions of the anterior-most structures.

6) Clutches from wild-type female can produce rare ventralized embryos.

The *hec^t2800^* allele was originally isolated in an early-pressure-based screen for recessive maternal-effect mutations [14], [17] and its effects described previously [20], [23]. Two additional alleles, *hec^p08ajug^* and *hec^p06ucal^* were identified in another maternal-effect mutant screen, based on a four-generation scheme [16], [18]. Using DNA markers linked to the *hec^t2800^* allele [20], we determined that the *hec^p08ajug^* and *hec^p06ucal^* alleles were linked to the same SSLP markers, z59658 and z24511 on chromosome 8. In addition, we carried out pair-wise crosses between individuals carrying the three mutant alleles to test for non-complementation. All crosses resulted in females that exhibited the *hec* mutant phenotype in their offspring in the expected proportions, i.e. Mendelian for F_1_ females (approximately 50% in crosses between homozygous males and heterozygous females of all allelic combinations) and maternal-dependent (near 100%) for F_2_ embryos (Table S1), indicating that all three mutations are part of the same complementation group.

A comparison of the phenotypes for the three *hec* alleles suggests that they fall within an allelic series. Embryos from mutant females carrying each of the three alleles were classified and scored at 24 hours post-fertilization (hpf; Table 1). The *hec^p06ucal^* allele shows the strongest average phenotype among the three alleles, where most embryos (76.3% from 3-month females) exhibit the strongest (V4) phenotype, while *hec^t2800^* mutants exhibit intermediate phenotype (46.7% V4 class) and *hec^p08ajug^* mutants show the weakest phenotype (10.9% V4 class). Double-axis embryos are observed only in offspring derived from females mutant for the two weaker alleles *hec^p08ajug^* and *hec^t2800^*, and only in clutches with weak penetrance and expressivity (Table 1 and data not shown). In the case of *ichabod* and *tokkaebi* mutations [5], [15] axis induction defects have been reported to vary with maternal age, with younger females exhibiting stronger phenotypes. We tested embryos from 3 month old and 12 month old mutant females and find a similar trend for the effects of the three *hec* alleles on axis induction (Figure S1).

Previous studies have shown that the ventralized phenotype of *hec* mutant embryos is associated with a reduction in the embryonic shield and changes in patterns of gene expression in dorsal- and ventral-specific genes in the late blastula embryo. Expression of gene markers of the various germ layers, such as *bmp2* and *gata2* (ectoderm), *no tail* (mesendodermal precursors), and *foxa2* (endoderm) was unaffected, other than regional differences due to predicted changes in dorsoventral specification [20]. As expected, embryos from females mutant for the newly isolated *hec* alleles, *hec^p06ucal^* and *hec^p08ajug^*, similar to the *hec^t2800^* allele [20], exhibit a reduction in the shield region corresponding to the dorsal organizer at the incipient dorsal region (Figure 1G–J), as well as a reduction in dorsal-specific gene expression (*goosecoid*, Figure 1K–N; *chordin*, Figure 1O–R) and a concomitant expansion of a ventrally-expressed gene (*eve 1*) (Figure 1S–V).

Together, phenotypic, linkage and complementation analysis indicate that these three mutations are alleles of the same gene.

### *hecate* encodes the zebrafish Glutamate receptor interacting protein 2a (Grip2a)

**Figure 2:**
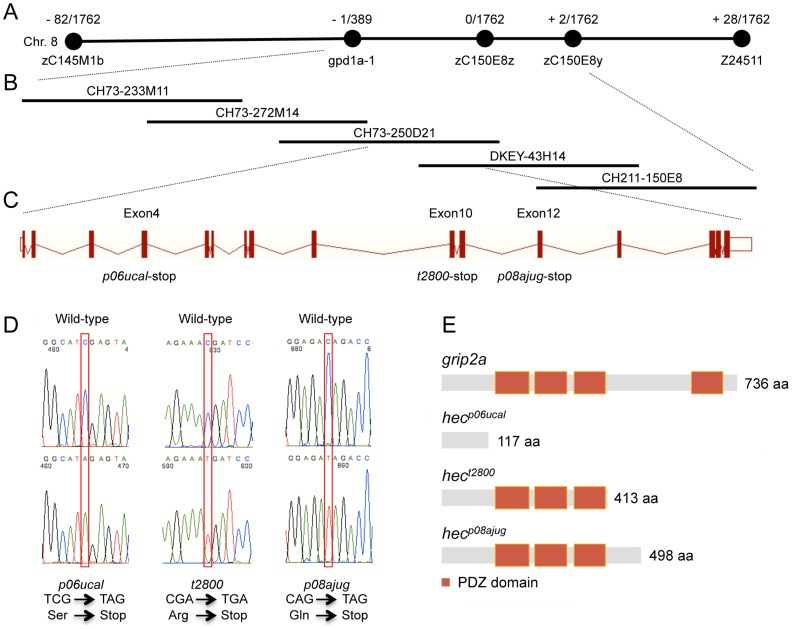
Molecular identification of the *hecate* locus. A) Linkage map of the *hec* locus. The number of recombinants over the total number of analyzed meiosis is indicated. *hec* linkage was initially identified between SSLP markers z59658 and z24511 on chromosome 8. Fine mapping analysis with newly identified RFLP markers further narrowed the region between the gene *gpd1a-1* and the RFLP zC150E8y. B) Contig of five BAC clones covering the *hec* critical region. CH73-233M11, CH73-272M14, CH73-250D21, DKEY-43H14 and CH211-150E8 are five sequenced and overlapping BAC clones in this interval. C) Exon-intron structure of the *hec/grip2a* gene, which contains 16 exons. The *hec^p06ucal^*, *hec^t2800^* and *hec^p08ajug^* alleles each cause a premature stop-codon in exon 4, exon 10 and exon 12, respectively. D) Sequence traces of the cDNA products from wild-type and the three mutant *hec* alleles. Nucleotide substitutions are indicated by the red box. Mutant cDNAs show a C-A transversion in codon 118 (*hec^p06ucal^*), a C-T transversion in codon 414 (*hec^t2800^*), or a C-T transversion in codon 499 (*hec^p08ajug^*), all creating premature STOP codons. E) Schematic diagram showing the protein domain structures of Grip2a in the wild-type and mutant alleles. Red boxes represent conserved PDZ domains.

To better understand the function of the *hec* gene, we determined its molecular identity using a positional cloning approach. We initially identified linkage of the *hec* locus between SSLP markers z59658 and z24511 on chromosome 8 through mapping of the *hec^t2800^* allele [20]. Homozygous mutant males were crossed to heterozygous females to generate large numbers of fish for fine mapping. Fine mapping analysis of 1762 meioses with newly identified RFLP markers further narrowed the critical region containing *hec* to a genomic region between *gpd1a-1* and the zC150E8y RFLP in the Ensembl database, corresponding to an interval of 383 Kb (Figure 2A). Five overlapping BAC clones were identified and aligned as a contig covering the whole critical region (Figure 2B). Within this critical region, there are 11 predicted genes according to the Ensembl database of the zebrafish genome and the GENSCAN program. Sequencing of cDNA products from wild-type and mutant alleles revealed the presence of mutations in all three *hec* alleles in the gene *glutamate receptor interacting protein 2a* (*grip2a*, NP_001116760.1), one of two *grip2* genes in the zebrafish genome [28].

The zebrafish *grip2a* gene has 16 exons, which produces a 3,124 bp transcript and a 736 amino acid protein (Figure 2C). The three mutant alleles result in truncated forms of the Grip2a protein: *hec^p06ucal^* allele has a C-A transversion in codon 118 in exon 4, *hec^t2800^* has a C-T transversion in codon 414 in exon 10, and *hec^p08ajug^* has a C-T transversion in codon 499 in exon 12, and these three mutations generate premature nonsense (stop) codons (Figure 2D). A search in the Conserved Domain Database (CDD) in NCBI [29] indicates that Grip2a protein has four PDZ domains. The premature stop-codons for these different alleles delete the most C-terminal PDZ domain in the *hec^t2800^* and *hec^p08ajug^* alleles, and all four PDZ domains in the *hec^p06ucal^* allele (Figure 2E). The retention of PDZ domains and size of the predicted truncated proteins roughly correlates with the observed phenotypic strength in the various mutant allele backgrounds (Table 1, Figure S1), although we have not determined expression levels for the mutant proteins to confirm their relative activities. The identification of mutations in these three independently isolated alleles indicates that *hec* encodes Grip2a. This is further substantiated by the localization of *grip2a* mRNA in the region of the embryo affected by the *hec* mutation (see below).

Using BLAST searches on Ensembl and NCBI genome databases, homologous *grip1* and *grip2* genes were found for all vertebrate species, such as fish, amphibians, birds, and mammals. In invertebrate lineages, a distantly related *Grip* gene was identified only in Drosophila. Grip1 and Grip2 protein sequences among eight representative species were used to construct a phylogenetic tree using ClustalW (Figure S2). *grip2* occurs as a single copy in amphibians, birds and mammals but is duplicated in the zebrafish and other fish species such as fugu and medaka, likely a consequence of an extra round of whole genome duplication in the ray-finned fish lineage [30], [31]. Drosophila and all vertebrate Grip1 and Grip2 proteins contain PDZ domains but zebrafish Hec/Grip2a and Fugu Grip2b contain 4 predicted PDZ domains instead of the 7 PDZ domains predicted in other members of this family (Figure S2 and not shown).

### *grip2a* mRNA is localized to the vegetal region of the oocyte, developing an early asymmetry upon egg activation

Quantitative RT-PCR analysis of mRNA from wild-type embryos spanning early development indicates highest levels of *grip2a* mRNA in the 1-cell stage embryo, gradually declining to negligible levels at 50% epiboly (5.25 hpf) and thereafter (Figure S3). In adults, expression can be detected in wild-type females and isolated ovaries, but not in males or female carcasses where the ovaries have been removed (Figure S3). Thus, at our level of analysis, *hec/grip2a* is specifically expressed in ovaries as a maternal-specific transcript, which is consistent with the strict maternal effect observed in females homozygous for the three *hec* mutant alleles.

**Figure 3:**
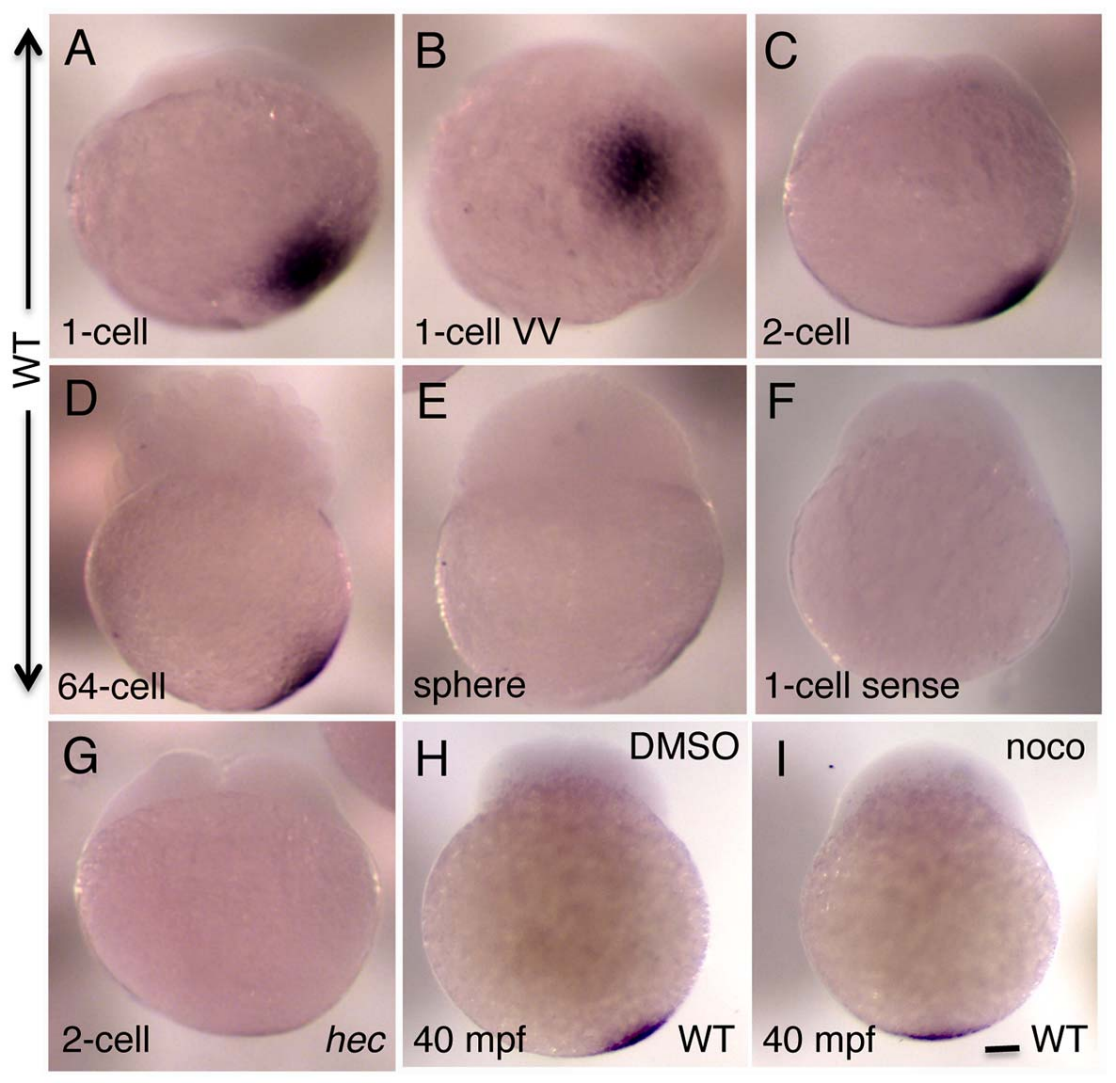
Whole mount in situ hybridization analysis of the expression of zebrafish *grip2a* mRNA in early embryos. A–E) *grip2a* mRNA is localized in a slightly off-center position at the vegetal pole of early embryos, and remains in that position until the sphere stage when it becomes undetectable. F) Control sense probe. G) The domain of *grip2a* mRNA localization in *hec* mutants is reduced in intensity and appears to lack an off-center shift. H,I) Representative control (DMSO)- and nocodazole-treated embryos showing the lack of off-center shift in nocodazole-treated embryos (see Figure S4 for details). All panels are side views (except for (B), which is a vegetal view of the embryo in (A)) at the following stages: A,B: 1-cell (20 mpf), C,F,G: 2-cell (45 mpf), D: 64-cell (2 hpf), E: sphere (4 hpf). (H,I) are at 40 mpf, which approximately corresponds to the 2-cell stage (C) in untreated embryos. Magnification bar in (I) corresponds to 100 µm for all panels.

We examined the spatial expression pattern of *hec/grip2a* at various developmental stages during embryogenesis using whole mount in situ hybridization (Figure 3). *grip2a* mRNA is detected in the vegetal pole region of the yolk in early zygotes and cleavage-stage embryos (Figure 3A–E). Similar to the case of *wnt8a* mRNA [7] and Sybu protein [6], the *grip2a* mRNA localization domain is not precisely aligned with the vegetal pole in activated eggs or early embryos. Instead, *grip2a* mRNA is consistently located slightly off-center in the post-activation stages examined, from the early 1-cell stage embryo 10 minutes post-fertilization (mpf) until late cleavage stages (Figure 3A–D). This off-center shift is not observed in manually extruded mature, inactive eggs, where the *grip2a* mRNA localization domain is instead located at the vegetal pole in a radially symmetric manner (data not shown). In early embryos the extent of asymmetry of the *grip2a* mRNA localization domain, appears similar throughout the cleavage and blastula stages until mRNA levels become markedly reduced in the late blastula embryo (sphere stage; 4 hpf; Figure 3E). Localized *grip2a* mRNA can no longer be detected starting at the onset of epiboly (30% epiboly; 4.66 hpf; not shown). In contrast to its *Xenopus* homologue [32], zebrafish *grip2a* mRNA does not localize to the zebrafish germ plasm (Figure 3C and data not shown), present at the furrows corresponding to the first and second blastomeric divisions [33]–[37], nor does it become incorporated into the primordial germ cells (Figure 3D,E and data not shown), which form four cell clusters during the late cleavage stages ([33], [35], [38]; see below).

To further confirm the slight, off-center shift in the *grip2a* mRNA localization domain, and to determine whether this shift, like that of Sybu protein and *wnt8a* mRNA, depends on an intact microtubule network, we tested the effect of early nocodazole treatment on the *grip2a* mRNA localization pattern in wild-type embryos. Embryos were treated at 5 mpf and fixed at 30 and 40 mpf for in situ hybridization. For both time points, control (solvent-treated) embryos show an off-center shift in the domain of *grip2a* mRNA localization so that it is located within an arc at 0–20° from the true vegetal pole of the embryo, while nocodazole-treated embryos do not exhibit a discernable shift in mRNA localization (Figure 3H, 3I; Figure S4). Thus, *grip2a* mRNA is located at the vegetal pole of the embryo in mature oocytes, but upon egg activation (typically coupled to fertilization) this mRNA exhibits a short-range, off-center translocation within the vegetal region of the embryo. Once in an off-center position *grip2a* mRNA remains static until its degradation at late cleavage stages.

Whole mount in situ hybridization of embryos from mutant female mothers homozygous for each of the three *hec* alleles show significantly reduced levels of localized *grip2a* mRNA during the early cleavage stages, ranging from reduced to nearly undetectable levels (Figure 3G and data not shown). In those embryos where *grip2a* mRNA localization can be discerned, the domain of localization appears centered at the vegetal pole, consistent with the absence of an off-center shift for vegetally localized products in *hec* mutants (see below). Quantitation of total *grip2a* mRNA levels in mutant embryos at the 2-cell stage indicates that, for all alleles, *grip2a* mRNA abundance is drastically reduced to approximately 15–25% that in wild-type embryos (Figure S3). It is possible that *hec/grip2a* function is required for the localization of its own mRNA, which when not localized is unstable. Alternatively, the reduction in apparent *grip2a* mRNA localization in *hec* mutant embryos might be a consequence of a decrease in *grip2a* mRNA abundance in these embryos, possibly by non-sense mediated mRNA decay as has been proposed for other maternal transcripts [39], [40].

### Vegetal localization of *grip2a* mRNA is initiated during oogenesis and depends on oocyte polarity genes

**Figure 4:**
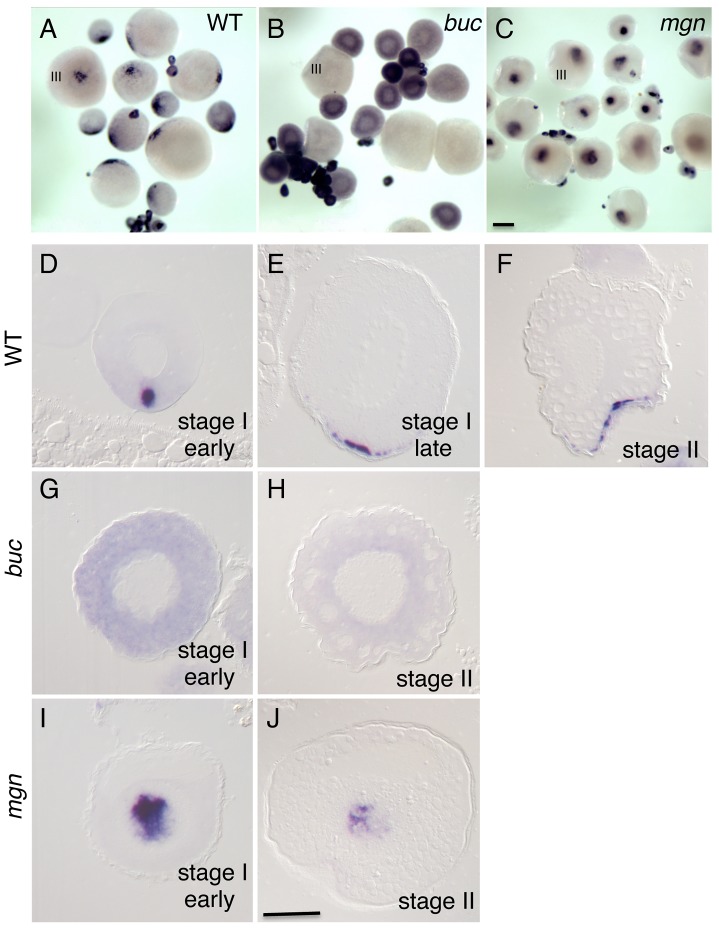
Localization of *grip2a* mRNA in wild-type and mutant oocytes. A–C) Whole mount in situ hybridization of dissected ovaries from wild-type (A), *bucky ball (buc*, B) and *magellan (mgn*, C) mutant females. In (A–C) oocytes at stage III of development are indicated. Smaller oocytes are at stages I and II, which are difficult to differentiate in whole mounts at this magnification. The *grip2a* mRNA localization domain is observed in an asymmetric cortical position in wild-type oocytes (A) but is unlocalized and diffuse in *buc* oocytes (B) and internally-located in *mgn* mutants (C). D–J) Sections of wild-type and mutant oocytes at the indicated stages after labeling to detect *grip2a* mRNA. Stages are as indicated in the panel and were determined by size and oocyte morphology according to [101]. D–F) Wild-type oocytes showing localization to the presumptive Balbiani Body (D) and subsequent localization to a cortical domain of the oocyte corresponding to the presumptive vegetal pole (E–F). G,H) *buc* mutant oocytes lack the Balbiani body [39], [43] and the *grip2a* mRNA subcellular localization domain in stage I and II oocytes. I) *mgn* mutant stage I oocytes exhibit an enlarged Balbiani body [40] and displayed an enlarged *grip2a* mRNA localization domain. J) Stage II mgn mutant oocytes fail to localize transcripts to the vegetal pole which instead persist in an internal domain [40], as observed also for *grip2a* mRNA. Number of oocytes examined were as follows: wild-type: early stage I: 13, stage II: 12; *buc*: early stage I: 35, stage II: 26; *mgn*: early stage I: 23, stage II: 18. Magnification bar in (C) corresponds to 250 µm for panels (A–C), and in (J) to 50 µm for panels (D–J).

To visualize how the pattern of *grip2a* mRNA localization is established during oogenesis, we carried out in situ hybridization analysis of wild-type oocytes at various stages using a *grip2a* antisense probe (Figure 4). In early stage I oocytes, *grip2a* transcripts appear sharply localized to a compact, spherical region at one region of the oocyte (Figure 4A, 4D). By late stage I, *grip2a* mRNA acquires a more spread, subcortical localization pattern still centered in one region of the oocyte (Figure 4A, 4E). This cortical localization is maintained until the end of oogenesis (Figure 4A, 4F and data not shown).

The localization pattern of *grip2a* mRNA in stage I oocytes is reminiscent of the zebrafish Balbiani body, a conserved aggregate of organelles present in animal oocytes shown to anchor subcellularly localized oocyte mRNAs [41]–[43]. We therefore tested whether *grip2a* mRNA localization is dependent on Balbiani body formation, using zebrafish mutants that affect this structure. Oocytes mutant for the gene *bucky ball* lack the Balbiani body [39], [43], and we found that *bucky ball* mutant oocytes lack *grip2a* mRNA subcellular localization during oogenesis (Figure 4B, 4G, 4H). Moreover, oocytes mutant for the cytoskeletal linker protein *magellan* (*macf1*), which exhibit an enlarged Balbiani body with an abnormal location [40], exhibit *grip2a* mRNA mislocalization (Figure 4C, 4I, 4J) similar to other Balbiani-localized transcripts. These results indicate that *grip2a* mRNA becomes localized to the vegetal cortex during oogenesis by a Balbiani body-dependent mechanism.

### *hecate/grip2a* is required for microtubule rearrangements at the vegetal pole

**Figure 5:**
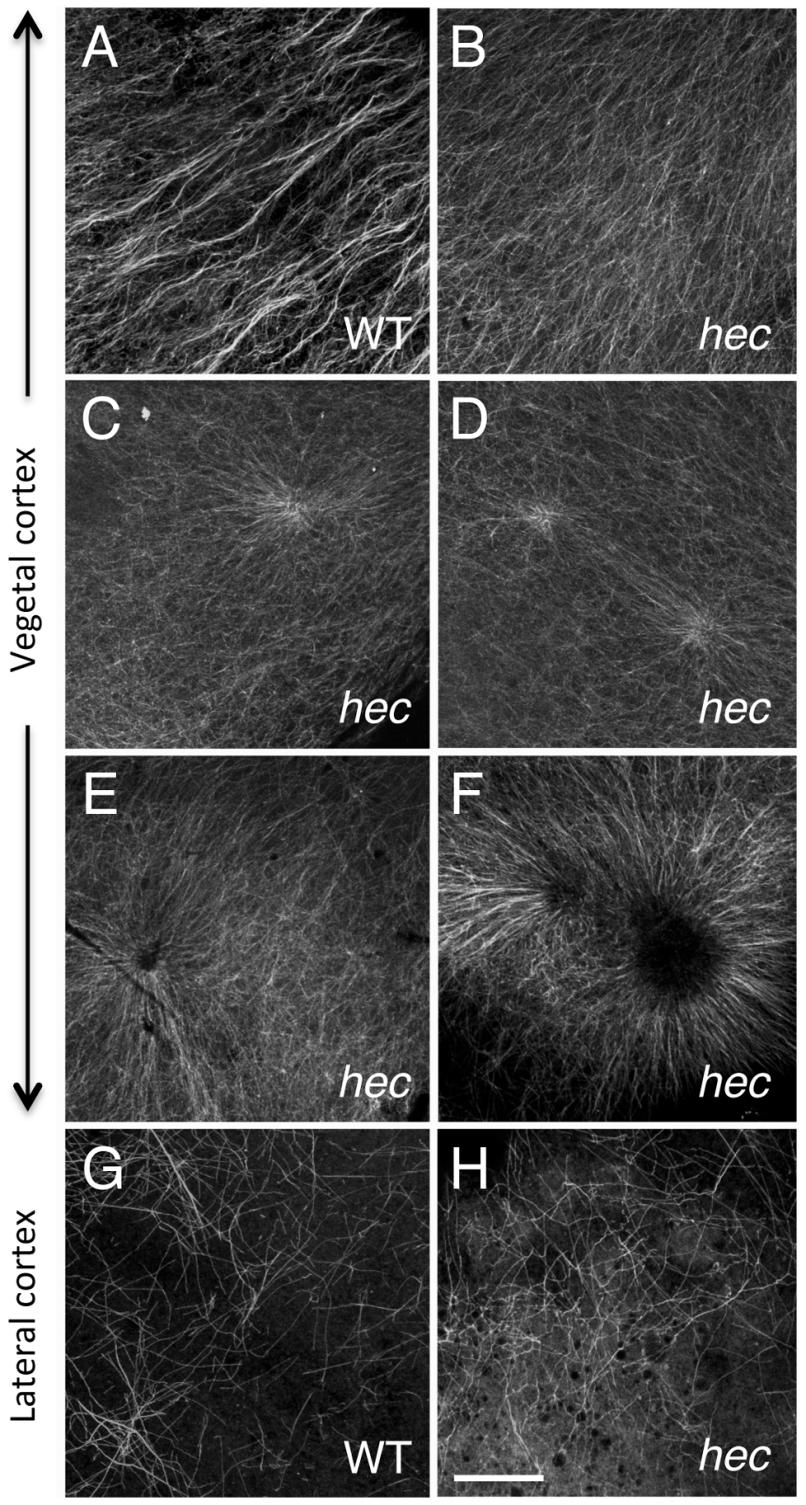
Microtubule reorganization at the vegetal cortex is affected in *hecate* mutants. A–F) Cortical microtubule network at the vegetal pole in wild-type (A) and *hec* mutant (B–F) embryos at 20 mpf. Microtubules appear oriented in the same direction and bundled in wild-type embryos (A). The extent of co-orientation and bundling is greatly reduced in *hec* mutant embryos (B), where microtubules form multiple aster-like structures which can have a well focused-center (C,D) or can exhibit a central microtubule-free zone (E,F) and often overlap (F) or interdigitate (D). The relatively unbundled microtubule arrangement shown in (B) also corresponds to a sector of a large aster-like structure emanating from a not shown central core. Up to 6 aster-like structures were observed in the vegetal cortex of a single embryo. G,H) Cortex in mediolateral regions shows a loose and apparently random network of microtubules which appears similar in both wild-type (G) and *hec* mutant (H) embryos (n = 8 for wild-type and mutants). All images are z-axis projections of confocal image stages. The phenotype was fully (100%) penetrant according to the two main categories (wild-type, aligned and bundled microtubules; mutant, radialy oriented and unbundled microtubules, with 10 wt and 25 *hec* mutant embryos imaged. Magnification bar in (H) corresponds to 40 µm for all panels.

The rescue of *hec* mutant embryos by overexpression of Wnt pathway components has suggested that *hec* likely activates signaling at an upstream step of the pathway [20]. Given that an early event in the pathway leading to Wnt signaling activation is the reorganization of microtubules at the vegetal pole required for the transport of local determinants, we tested whether this reorganization is affected in *hec* mutant embryos (Fig. 5; Figure S5). Consistent with previous studies [2], [8], [27], we find that microtubules at the vegetal cortex in wild-type appear as parallel tracks of bundled microtubules at 20 mpf (Figure 5A). In *hec* mutant embryos, such parallel tracks of bundled microtubules are not observed (Figure 5B). In these mutants, unbundled microtubules typically appear to radially emanate from one or more aster-like structures at the vegetal pole region (Figure 5C–F). Exposure to the microtubule-stabilizing drug taxol [44] during the first cell cycle (5 to 35 mpf) does not influence the degree of residual axis induction in *hec* mutants (Figure S6), suggesting that the observed defects may not be simple consequence of altered microtubule dynamics. Labeling of the F-actin cortical network in the vegetal cortex region shows a similar appearance in wild-type and mutant embryos (Figure S7), including the presence of F-actin rich protrusions as previously described [45].

### Short-range symmetry breaking and anchoring of vegetally localized factors are affected in *hec* mutant embryos

**Figure 6:**
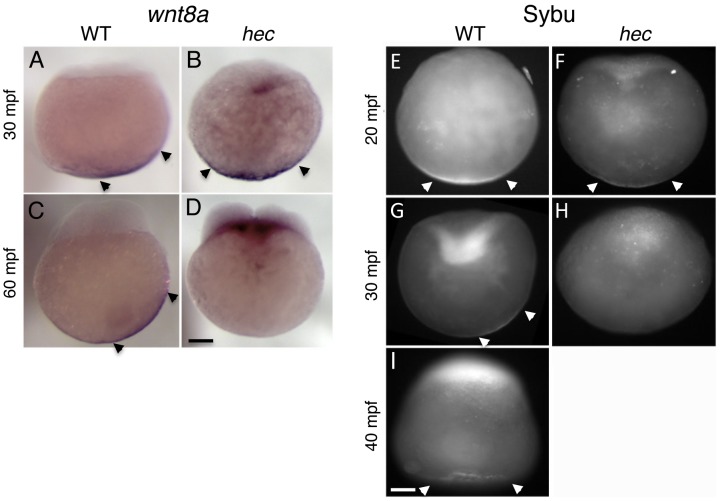
Defects in the vegetal localization of *wnt8a* mRNA and Sybu protein. A–D) Off-center shift of *wnt8a* mRNA is affected in *hec* mutants. Whole mount in situ hybridization of wild-type embryos (A,C) and *hec* mutant embryos (B,D) at the 1- (A,B, 30 mpf) and 4- (C,D, 60 mpf) cell stages. Images show representative embryos. A majority of wild-type embryos showed a clear off-center shift (85%, n = 27 at 30 mpf and 74%, n = 47 at 60 mpf). A majority of *hec* mutant embryos showed vegetal localization without a shift at 30 mpf (79%, n = 33, remaining embryos show no localization) and absence of localization at 60 mpf (89%, n = 38, remaining embryos show reduced vegetal localization without a shift). The apparent label at the base of the blastodisc is observed in a majority of mutant embryos (71%, n = 38) but not in wild-type (C) or control embryos labeled with other probes (not shown) and may reflect remaining *wnt8a* mRNA that has lost anchoring at the vegetal pole and has moved animally through the action of axial streamers [83]. E–I) Localization of Sybu protein is affected in *hec* mutants. Whole mount immunofluorescence to detect Sybu protein of untreated wild-type (E,G) and *hec* mutant (F,H,) embryos and nocodazole-treated wild-type embryos (I) at the indicated stages. In wild-type embryos, an off-center shift in Sybu protein can be observed starting at 30 mpf (G). In *hec* mutants, Sybu protein becomes undetectable levels by this same time point (H). Patterns of localization of Sybu protein at 10 mpf and 20 mpf time points (combined n: 32 WT, 19 mutant for 10–20 mpf), and 30 mpf and 40 mpf time points were similar and have been combined. 59% (n = 32) of wild-type and 63% (n = 19) of *hec* mutant embryos showed centered vegetal localization during 10–20 mpf. At 30–40 mpf, the percent of embryos that showed vegetal localization, now with an off-center shift, was reduced to 25% (n = 28) in wild-type, and 0% (n = 25) of *hec* mutants showed any localization at these time points. Treatment of wild-type embryos with nocodazole inhibits the shift but does not result in delocalization from the vegetal cortex (I, embryo at 40 mpf), as previously shown [6]. Magnification bars in (D) and (I) correspond to 100 µm for panels sets (A–D) and (E–I), respectively.

Since vegetal cortical microtubules have been proposed to mediate the off-center shift of factors involved in axis induction that are initially localized to the vegetal pole, such as *grip2a* mRNA (this report), *wnt8a* mRNA ([7]; Figure 6A, 6C) and Sybu protein ([6]; Figure 6E, 6G), we tested whether these shifts were affected in *hec* mutant embryos. As noted above the *grip2a* mRNA localization domain in *hec* mutants, when still detectable, fails to undergo an off-center shift (Figure 3G). In situ hybridization analysis to detect *wnt8a* mRNA shows this mRNA also fails to undergo a noticeable off-center shift in one-cell (30 mpf) *hec* mutant embryos (Figure 6B). At the 4-cell stage (60 mpf), *wnt8a* mRNA localization at the vegetal pole is significantly reduced or undetectable (Figure 6D), although this defect is associated with an overall decrease in the relative expression of *wnt8a* mRNA (Figure S8). In the case of Sybu protein, wild-type embryos show localization centered at the vegetal pole until 20 mpf (Figure 6E) and an off-center shift of protein localization by 30 mpf (Figure 6G), as previously reported [6]. In *hec* mutants, the Sybu protein localization domain can be initially detected centered at the vegetal pole of *hec* mutants (Figure 6F). However, Sybu protein is no longer detectable by 30 mpf (Figure 6H), precluding testing an effect on Sybu protein off-center movement. These data indicate that *hec/grip2a* is essential for the short-range, symmetry-breaking transport of vegetally-localized factors, and are consistent with *hec* function being essential for microtubule reorganization in this region. The reduction in vegetally localized *wnt8a* mRNA and Sybu protein *in hec* mutants contrasts with the perduring vegetal localization of these factors in embryos with a perturbed microtubule network ([6], [7]; Figure 3I, 6I).

### Long-range animally-directed transport is independent of *hecate/grip2a* function and not restricted to the prospective dorsal region

The short-range off-center shift observed in the case of Sybu protein and *grip2a* mRNA, which occurs within the confines of the vegetal region, contrasts with the long-range transport thought to be involved in transporting a putative dorsal signal to blastomeres at the animal region [2]. *wnt8a* mRNA has been observed to reach the base of the blastomeres by the 16-cell stage (1.5 hpf; [7]), although in our experiments this RNA exhibits a relatively static off-center shift throughout the first 60 mpf (Figure 6A, 6C), similar to the short-range movement of Sybu protein and *grip2a* mRNA. The animally-directed translocation of a putative dorsal signal is thought to be reflected in the microtubule-dependent, animally-oriented movement of small (0.2 µm) polystyrene fluorescent beads during the first several cell cycles. When injected into the vegetal region, these beads reach the base of the blastomeres at the animal region by traveling through cortical paths [2].

**Figure 7:**
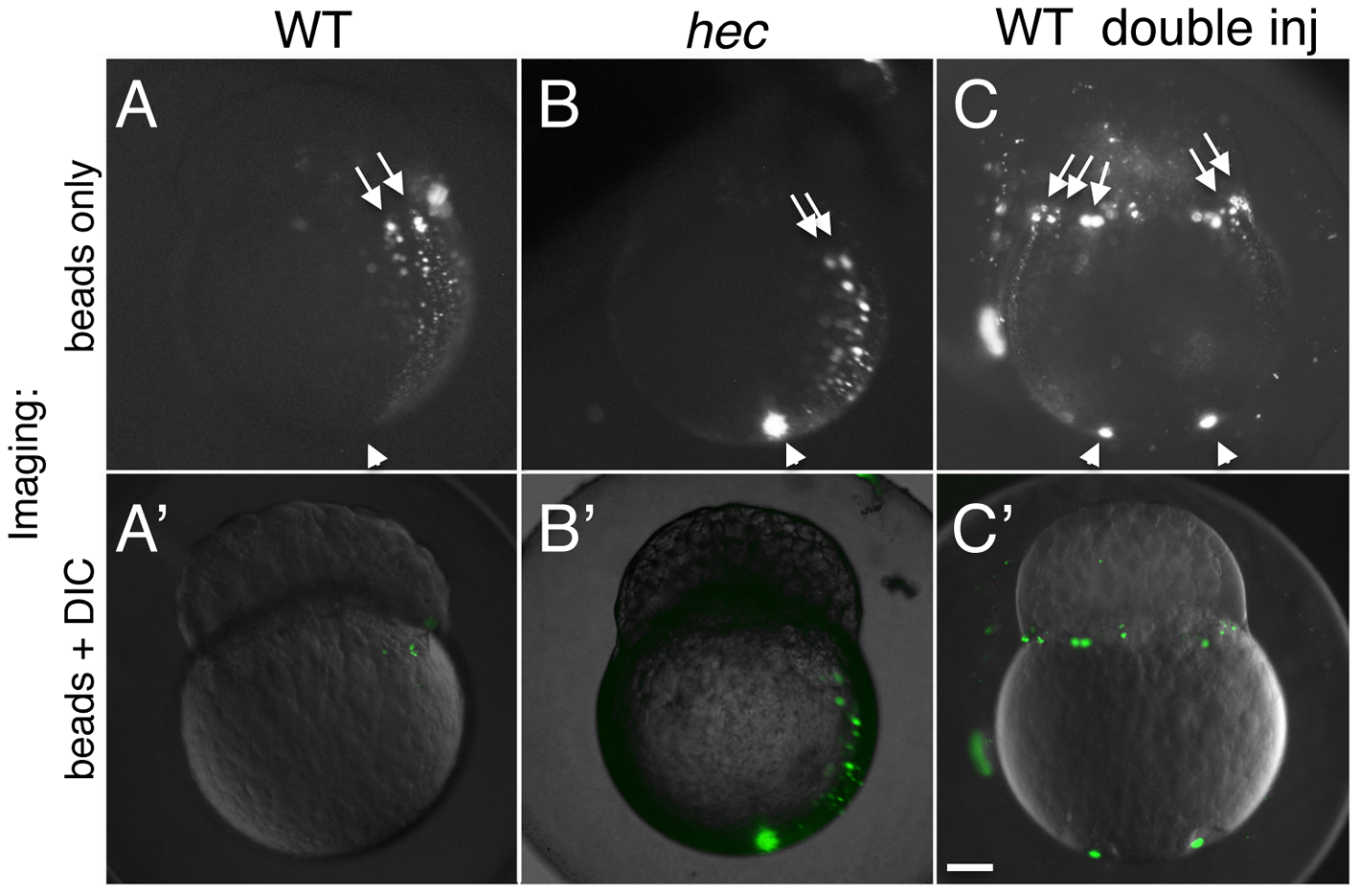
Long-range animally-directed transport is not affected in *hecate* mutants. A–C) Paths of injected 0.2 um fluorescent beads after injection into the vegetal pole with a single (A,B) or double (C) injection in wild-type (A,C) or *hec* mutant (B) embryos. (A′–C′) show merged imaged including the fluorescent channel (shown in A–C) and corresponding DIC optics at low intensity. The extent and frequency of bead transport appeared similar in wild-type and mutant embryos (A,B, see text). Injections into two opposite sides of the vegetal pole results in multiple animally-directed paths, indicating that the entirety of the mediolateral cortex is competent for bead movement. Arrowheads and arrows in (A–C) indicate site of injection in the vegetal region and animally-directed paths along mediolateral regions, respectively. Magnification bar in (C′) corresponds to 100 µm for all panels.

Using the transport of microinjected fluorescent beads as an assay for this long-range transport mechanism, we tested whether long-range vegetal-to-animal movement along the cortex might be affected in *hec* mutant embryos. As previously reported [2], in wild-type embryos bead movement from the vegetal region is observed along a meridional arc along the cortex reaching the base of the blastomeres at the animal pole (41% (n = 87); Figure 7A,A′). In *hec* mutant embryos, beads appear to be transported to a similar extent as in wild-type, also reaching the base of the blastodisc (Figure 7B,B′) and at a similar observed frequency (39% (n = 51)). Thus, *hec* function does not appear to be required for long-range animally-directed transport along the lateral cortex. The apparently normal movement of animally-directed beads in *hec* mutants appears to conflict with the role of this gene in early microtubule reorganization but is consistent with the observed presence of multiple microtubule populations [2], [8]: aligned bundles of short microtubules at the vegetal region, which we find to be dependent on *hec* function, and a more dispersed and randomly oriented network in more medial regions, which appears unaffected in *hec* mutants (Figure 5G, 5H and data not shown).

The direction of aligned short microtubule bundles at the vegetal pole has been shown to correlate with the dorsal axis but only occurs in a limited area within the vegetal half of the early embryo [8]. Given that directionality of a long-range movement might depend on an earlier oriented symmetry-breaking event, we wondered whether the long-range animally directed movement was specific to the putative dorsal region, or whether the entirety of the cortex was competent to support such transport. To test whether long-range animally-directed transport was specific to the prospective dorsal region of the embryo or was a general property of the lateral cortex, we carried out two slightly off-center fluorescent bead injections on opposite sides of wild-type embryos. Such doubly-injected embryos show animally-directed bead transport along meridional arches in opposite regions of the embryo (Figure 7C,C′), an observation inconsistent with only the prospective dorsal region mediating long range vegetal-to-animal cortical transport. Instead, our data suggest that the entirety of the cortex can mediate long-range animally-directed movement.

The apparently normal long-range transport of beads in *hec* mutant embryos, in the presumed absence of an early short-range symmetry-breaking process, may reflect asymmetries in the location of the beads during injection, followed by the action of this *hec*-independent long-range transport mechanism. Our data suggest that the transport of dorsal determinants to the prospective site of dorsal induction depends on two sequential processes: (i) an initial short-range transport dependent on *hec*-mediated formation of short aligned microtubule bundles that results in determinant asymmetry at the vegetal region of the embryo, and (ii) a subsequent long-range transport through lateral cortical regions, which is independent of *hec* function and not specific to the prospective dorsal region.

### Divergence of *grip* function in zebrafish and *Xenopus*

**Figure 8:**
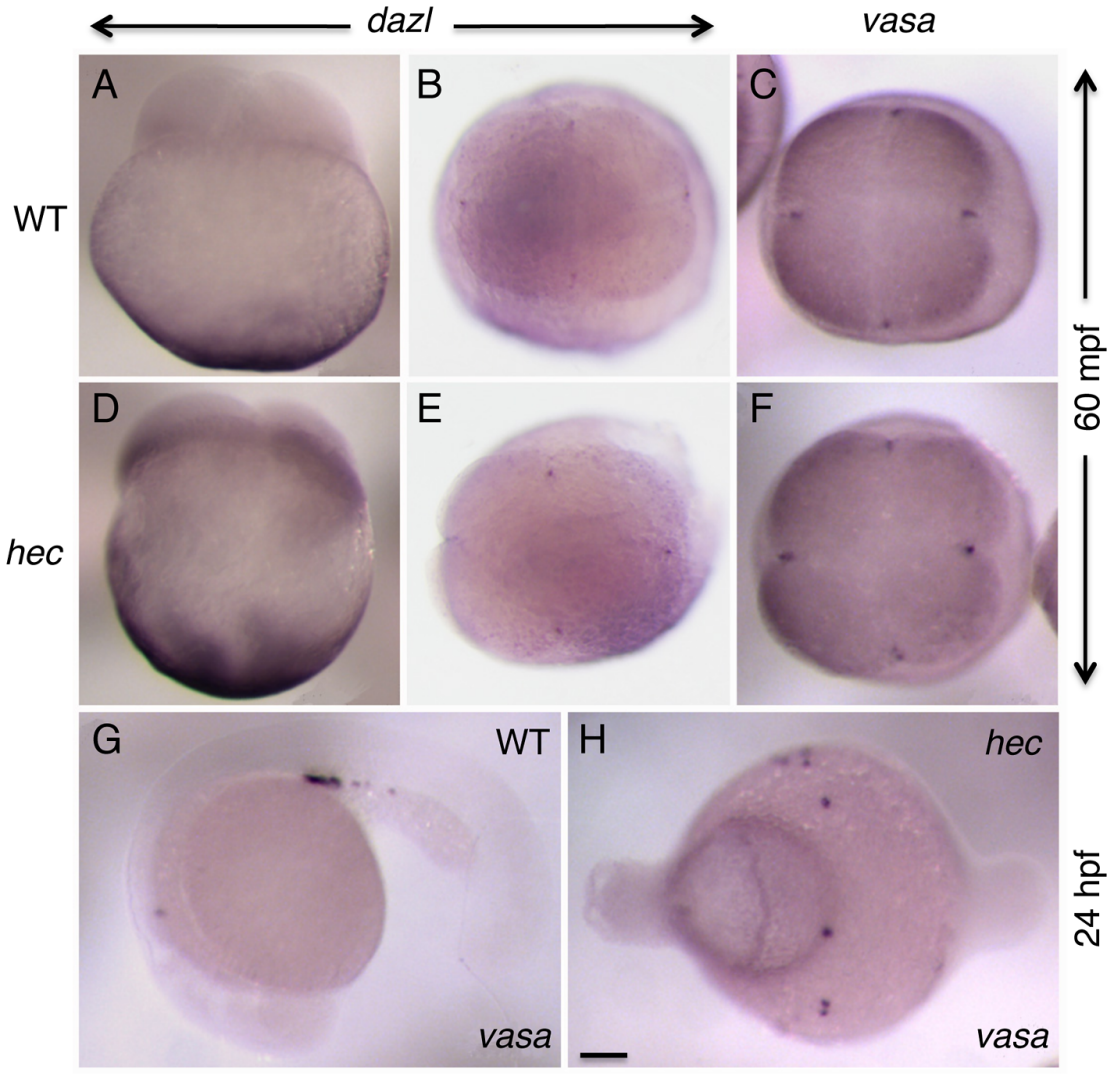
Germ plasm recruitment and PCG determination appears unaffected in *hecate* mutants. A–F) Whole mount in situ hybridization of 4-cell stage (60 mpf) embryos to label the germ line-specific genes *dazl1* (A,B,D,E), and *vasa* (C,F) in wild-type (A–C) and *hec* mutant (D–F) embryos. Early localization of *dazl* mRNA to the vegetal pole (A,D, side views), and *dazl* mRNA and *vasa* mRNA to the germ plasm as it becomes recruited to the furrows of the first and second cleavage cycles (B,C,E,F, animal views). Localization of dazl domains of recruitment at the furrow is not detected in the side views as the levels of mRNA in these domains are relatively low and the focal plane is not optimal for their visualization. G,H) PGC determination, as determined by *vasa* expression in 24-hour embryos (G), appears unaffected in *hec* mutants (H), although the PGCs in mutant embryos do not become clustered as in wild-type due to the aberrant cell specification in these mutants. Quantification of the number of *vasa*-expressing cells is presented in Figure S9. Magnification bar in (H) corresponds to 100 µm for all panels.

In *Xenopus*, the homologous gene *grip2* (previously referred to as *grip2.1*), like zebrafish *hec/grip2a*, is expressed maternally and its mRNA is localized to the vegetal pole of the egg and early embryo [32]. Following this vegetal localization, *Xenopus grip2* mRNA becomes incorporated into primordial germ cells (PGCs) where it plays a role in their migration and survival [32]. In contrast, although localized to the vegetal pole, zebrafish *grip2a* mRNA does not localize to the zebrafish germ plasm or PGCs (Figure 3 and data not shown). We used whole mount in situ hybridization to test whether germ plasm localization or PGC development may be affected in *hec* mutant embryos (Figure 8). The localization patterns of *dazl* mRNA, a germ plasm component initially localized to the vegetal pole of the egg ([36], [46], [47]; Figure 8A), is not affected in these mutants (Figure 8D). During the first two cell cycles, *dazl* mRNA localizes normally to the furrows in *hec* mutants (Figure 8B, 8E), as does *vasa* mRNA (Figure 8C, 8F), an animal germ plasm component already present in the animal cortical region during egg activation [33], [36], [38], [48]. During embryonic development, although PGC migration is abnormal in strong *hec* mutants due to their radially symmetric, ventroposteriorized morphology, the average number of induced PGCs (as determined by cells expressing *vasa* in the 10.5 hpf embryo [33], [38], [48]) is similar to that in wild-type embryos (Figure 8G, 8H; Figure S9). Thus, as opposed to the case of *Xenopus grip2*, our observations do not support a role for zebrafish *hec/grip2a* in PGC development.

Two other zebrafish *grip*-related genes, *grip1* and *grip2b*, exhibit maternal expression as assayed by RT-PCR of RNA from 1–4 cell embryos but do not show localization to the vegetal pole or germ plasm at these early stages, or in PGCs in the 24-hour embryo ([28]; our own data). Together, these data indicate that, in spite of similar mRNA localization patterns at the vegetal pole of the egg, *grip* homologues in *Xenopus* and zebrafish have divergent roles in PGC development and axis induction, respectively.

## Discussion

A gynogenesis-based forward genetic screen in the zebrafish led to the isolation of a mutation in *hec*, which results in axis induction defects [17], [20], [23]. Here, we report the isolation of two additional alleles of *hec*, identified through an F3 inbreeding screen, and the molecular identification of this gene as encoding Glutamate receptor interacting protein 2a. This molecular assignment, as well as the localization pattern of its products, suggests that vegetally localized *grip2a* mRNA in zebrafish acts at the vegetal pole in the early events of dorsal axis induction. Accordingly, we find that *hec* functions to reorganize and align microtubule bundles that are involved in the symmetry-breaking transport of factors localized to the vegetal cortex, which is essential for axis induction.

### Role of *hecate*/*grip2a* in cortical microtubule rearrangements and axis induction

In both teleosts and amphibians, dorsal determinants are initially localized at the vegetal pole and then translocate to the prospective dorsal side in a microtubule-dependent manner to initiate the dorsal cell fate program (reviewed in [1], [49], [50]). Genetic and molecular searches have identified factors localized to the vegetal cortex with a role in axis induction in the zebrafish, such as the kinesin-1 linker Syntabulin [5], [6] and the *wnt8a* mRNA [7]. We show that the mRNA product for zebrafish *hec/grip2a* is another zebrafish maternal factor involved in axis induction, whose product is also localized to the vegetal pole of the oocyte and early embryo.

Zebrafish *hec/grip2a* is expressed solely during oogenesis as a maternal transcript, which is consistent with the identification of multiple maternal-effect mutant alleles of this gene, all of which lack associated zygotic defects. In particular, fish homozygous for the *hec^p06ucal^* allele, predicted to lack all four PDZ domains present in the wild-type protein and therefore likely a null, exhibit a highly penetrant maternal-effect phenotype yet are themselves viable. These observations indicate that *hec/grip2a* has a dedicated function in early axis determination. While we cannot rule out that *hec/grip2a* is expressed in older embryos or adults at levels below the sensitivity of our assays, other related genes such as *grip1* or *grip2b* are expressed at later stages of development ([28]; our own data) when they may provide essential zygotic functions.

Mechanisms inducing microtubule bundling and alignment, essential for the establishment of the primary body axis, remain incompletely understood. In *Xenopus*, cortical rotation and microtubule reorganization are dependent on both kinesin and dynein motor activity [51], [52] and other factors such as Trim36, a ubiquitin ligase whose mRNA is localized to the vegetal egg cortex [53], Dead end, an RNA binding factor needed for *trim36* mRNA vegetal cortex localization [54] and the lipid droplet component Perilipin 2, whose mRNA is also localized to the *Xenopus* vegetal pole [55], [56]. We find that zebrafish *hec/grip2a* function is required for the reorganization of vegetal cortex microtubules into bundles normally directed towards the prospective dorsal axis [2], [8]. The lack of microtubule network alignment in *hec* mutants and associated defects in the transport of putative dorsal determinants such as *wnt8a* mRNA likely result in the axis induction defects observed in these mutants.

Grip was originally identified as a factor interacting with AMPA-type glutamate receptors [57] and its multiple PDZ domains are thought to facilitate protein-protein interactions within large macromolecular complexes, including the surface presentation and trafficking of transmembrane proteins [58]–[60]. *grip* genes have been implicated in epithelial development in both mouse and zebrafish embryos ([28], [61], reviewed in [62]). Other studies have implicated Drosophila Grip as a mediator of Wnt ligand activity in the postsynaptic terminal of the neuromuscular junction [21], [22].

Our findings suggest parallels between subcellular transport at the vegetal pole of the zebrafish zygote and transport of neurotransmitter receptors in neurons. In the zebrafish zygote, transport of *wnt8a* mRNA depends on microtubules and occurs concomitantly with the movement of the kinesin adaptor Syntabulin [6], [7]. Similarly, in dendrites Glutamate receptors associated with mammalian GRIP1 are driven by kinesin along microtubules [63], and Syntabulin has been shown to be required for axonal transport [64]–[66]. In neurons, glutamate receptors and GRIP associate with membrane vesicles [67], [68]. Although membrane vesicles have not been reported to be associated with dorsal determinants in zebrafish, studies in *Xenopus* implicate membrane vesicles in the transport of dorsal determinants [69]–[72]. Further studies will be required to determine mechanisms driving the reorganization of vegetal cortex microtubules, the precise role of Grip2a in this process and whether Grip factors have an analogous cytoskeletal restructuring function in other systems.

### Is Grip2a required for downstream events in Wnt signaling involved in axis induction?

Our previous studies have shown that manipulations to activate Wnt signaling, including the overexpression of *wnt8* mRNA, can rescue the *hec* mutant phenotype, which suggests that *hec* acts in an upstream event required for Wnt signaling activation during axis induction [20]. Our identification of a role for Grip2a on cytoskeletal events needed for the relocation of dorsal determinants is consistent with such an upstream role. However, our studies do not rule out a more direct role for Grip2a as a regulator of Wnt pathway components. We note that the off-center shift of vegetally localized *grip2a* mRNA upon egg activation could provide an asymmetric source of Grip2a protein to influence Wnt signaling activity at the prospective dorsal region. Drosophila Grip is known to interact with the Wnt receptor Frizzled-2, promoting the trafficking of a Frizzled-2 C-terminal fragment to the nucleus to activate target genes [21], [22], [73], [74], and it is possible that some of these interactions are conserved in the zebrafish embryo. It is also possible that Grip2a regulates non-canonical Wnt signaling, such as Wnt/calcium signaling, which in turn influences axis induction. *hec* mutants exhibit an increased frequency of intracellular calcium transients in blastula stage embryos (2.00–3.33 hpf; [20]), and the resulting intracellular calcium increase has been proposed to attenuate Wnt/β-catenin signaling pathway activity [75]–[79]. Further studies will be necessary to determine whether *hec/grip2a*, in addition to functioning in cytoskeletal organization in the early zebrafish embryo, has a direct role in the regulation of Wnt/β-catenin signaling and axis induction.

### A multiple-step mechanism for the transport of dorsal determinants in zebrafish

Multiple studies in *Xenopus* have indicated the formation of long tracks of cortical microtubules associated with the cortical rotation [80]–[82]. While in this organism the cortical rotation involves the concerted movement of the cortex along a distance corresponding to a 30° arc, microtubule tracks and so-called fast transport of subcellular components, such as membrane organelles and specific factors, encompass a longer distance corresponding to a 60–90° arc (reviewed in [1]). Thus, in *Xenopus* both the cortical rotation and fast transport may participate in the relocation of dorsal determinants. In zebrafish embryos, a cortical rotation-like process results in the displacement of granules along a 20° arc from the vegetal pole of the embryo [8], with mediolateral regions of the cortex exhibiting a loose meshwork of microtubules independent of dorsoventral position [2], [8]. The restriction of microtubule bundling and alignment to the vegetal region of the zebrafish embryo raises the question of how long-range transport of dorsal determinants to the animal pole is achieved.

We found that injected beads reach the base of blastomeres in *hec* mutant embryos, which lack a cortical rotation-like movement, with a frequency similar to that observed in wild-type embryos. This suggests that transport of beads along the mediolateral cortex is independent of *hec/grip2a* function and aligned vegetal microtubules. Our finding that beads are able to move animally along opposite sides in multiply injected embryos, further suggests that the entirety of the mediolateral cortex, not just the prospective dorsal region, is competent for long-range vegetal-to-animal transport. Previous studies have identified animally-directed transport movement of cytoplasmic particles along cortical “meridional” streamers, hypothesized to mediate transport of vegetally-injected fluorescent beads [83]. This meridional transport along the mediolateral cortex may depend on various possible structures or processes, such as perpendicular bundles aligned along the animal-vegetal axis in deep regions of the cortex [8], incipient yolk cytoplasmic layer (YCL) microtubules emanating from marginal blastomeres into the yolk [84], an emerging property of the loose network of cortical microtubules found in this region of the embryo [2], [8], or other insofar unidentified cytoskeletal networks. Further analysis of the dynamic aspects of cytoskeletal networks in this region will be required to understand the mechanistic basis of this meridional transport system and its role in axis induction. In addition, our results do not exclude the possibility that diffusion of a translated protein such as Wnt8a may also contribute to long-range transport, as previously suggested [8].

**Figure 9:**
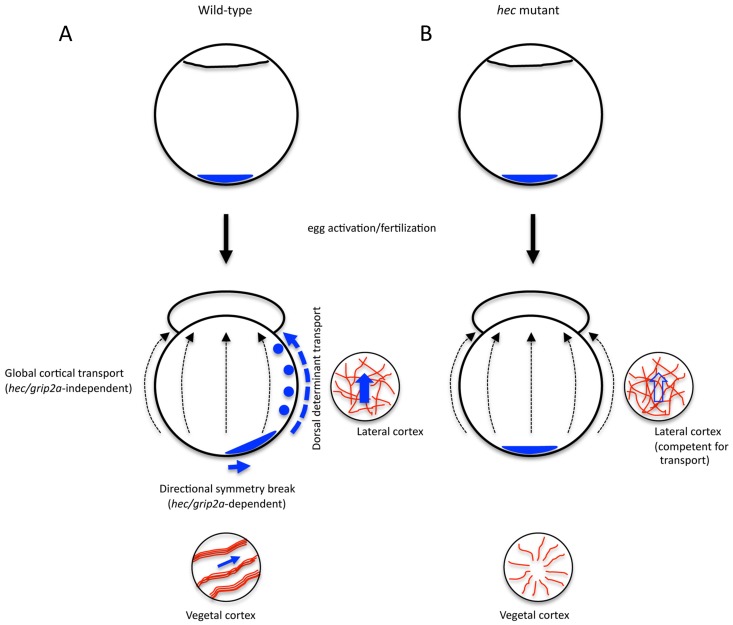
Amplification of *hecate/grip2a*-dependent symmetry breaking event by a general animal-directed long-range transport system. A) Cortical shifts of various vegetally localized components, including *wnt8a* mRNA, Sybu protein and *grip2a* mRNA ([6], [7], [8]; this report) are short-range and dependent on microtubule bundling and alignment, itself dependent on *hec* function. In wild-type embryos, such a short-range shift generates a symmetry breaking event that is subsequently amplified by long-range, animally-directed transport mechanism independent of *hec* function and not restricted to the prospective dorsal axis. B) In *hec* mutant embryos, neither reorganization of vegetal microtubules into aligned bundles nor a short-range shift occur, so that, even though long-range transport remains intact, vegetal determinant transport to the animal pole is affected. The mechanistic basis for the long-range transport, occurring in the region of a loosely organized mediolateral microtubule cortical network remains to be determined (see Discussion).

Together, these data indicate that the transport of the putative dorsal determinant in the early zebrafish embryo involves at least two separate mechanisms: a short-range transport dependent on *hec/grip2a* function and aligned microtubule bundles, and a subsequent long range transport relying on the mediolateral cytoskeletal network (Figure 9A). We hypothesize that the former generates an off-center, symmetry-breaking shift in initially symmetrically localized putative dorsal determinants, while the latter acts as a more general conduit that amplifies the early asymmetry. In *hec* mutants, the initial symmetry-breaking event is affected, so that even with a functional long-range animal transport mechanism, in most embryos an insufficient amount of dorsal determinants reaches the blastomeres at the animal pole (Figure 9B). A dual mechanism of dorsal determinant transport may also explain how a fraction of embryos from females homozygous for the presumptive null allele, *hec^p06ucal^*, which lacks all 4 conserved PDZ domains, can develop a normal dorsal axis. In such embryos small fluctuations may occur in the position of the vegetally localized dorsal determinant, which could be amplified by the mediolateral transport system that is unaffected in these mutants. The presence of an embryo-wide pathway directing long-range transport towards the animal pole may also explain the appearance of double-axis embryos observed only in the weakest *hec* mutant clutches. In these cases, an aberrantly organized vegetal microtubule network may result in the off-center vegetal shift of dorsal determinants in more than one direction, leading to their animally-directed transport along multiple paths and resulting in supernumerary or expanded regions of axis induction. A wider distribution of dorsal determinants in weak *hec* mutants would result in their reduced concentration in animal regions and reduced Wnt pathway activation, consistent with the observed lack of anterior-most structures in the resulting double-axis embryos. This dual transport model suggests mechanisms by which small, directed changes, like the specific early short range symmetry-breaking event, can be amplified during early development by an embryo-wide mechanism to result in large differences in cell fate specification.

### *hecate* may represent a gene duplication adapted for axis induction

Using BLAST searches on Ensembl and NCBI genome databases, homologous *grip1* and *grip2* genes were found for all vertebrate species. In Drosophila, there is an ancient single *Grip* gene. All other species containing recognizable *grip* genes are vertebrates. In amphibians, birds and mammals, there is one *grip1* gene and one *grip2* gene. On the other hand, zebrafish have one *grip1* gene and two *grip2* genes, likely due to an ancestral genome duplication in the teleost lineage [30], [31]. Following published nomenclature [28], we refer to the *grip2* gene corresponding to *hec*, located in chromosome 8, as *grip2a*, and the copy located in chromosome 22, as *grip2b*. Phylogenetic analysis indicates that zebrafish *grip2a* is only present in fish species (zebrafish, fugu and medaka). *Drosophila* Grip and all vertebrate Grip1 and Grip2 proteins (including zebrafish Grip2b) contain seven conserved PDZ domains, while zebrafish Grip2a contains only 4 PDZ domains.

The amphibian *grip2* homolog was identified as a novel vegetally-localized mRNA in *Xenopus* oocytes that is present in the mitochondrial cloud (Balbiani body) and subsequently in the germ plasm throughout oogenesis and early embryogenesis [32], [85], [86]. In *Xenopus grip2* morphants, PGC numbers are significantly reduced, and PGCs are also present at ectopic locations along the anteroposterior axis in tailbud stage embryos [32], [86]. However, unlike the case of *Xenopus grip2*, zebrafish *grip2a* mRNA does not localize to the germ plasm or PGCs, and the number of PGCs is unaffected in strongly ventralized mutants (i.e. radially symmetric ventralized embryos). These observations suggest that in zebrafish PGCs are determined independently of *hec/grip2a* function. Therefore, it appears that *Xenopus grip2* and zebrafish *grip2a* mRNA, in spite of a similar localization at the vegetal pole of the oocyte, have distinct functions in early embryogenesis: *Xenopus grip2* is involved in PGC development, while zebrafish *grip2a* appears to be devoted to dorsal axis formation.

Despite this divergent function, the fact that zebrafish *hec/grip2a* and *Xenopus grip2* are maternally expressed and localized to the vegetal pole during oogenesis, the site of localization of both germ plasm components and dorsal determinants in these two lineages, suggests that an ancestral *grip* gene may have functioned in both axis induction and PGC development. There is precedent for a relationship between these two processes, notably in Drosophila [87] but also in systems as basal as planaria [88], [89] and annelids [90]. This relationship is also supported by the recent finding that the germ cell-specific factor Dead end is required for microtubule rearrangements and cortical rotation in *Xenopus* [54]. The presence of a single localization system involved in both the induction of the primary embryonic axis and the separation between germ cell and soma may constitute a simple mechanism for the species patterning and propagation. Studies of the role of *grip* genes in other organisms may shed light on the relationship between axis induction and PGC determination during evolution.

## Materials and Methods

### Ethics statement

All animals were handled in strict accordance with good animal practice as defined by the relevant national and/or local animal welfare bodies, and all animal work was approved by the appropriate committee (University of Wisconsin – Madison assurance number A3368-01).

### Fish maintenance and genetic lines

Fish stocks were raised and maintained under standard conditions at 28.5° [91]. The *hec^t2800^* allele was originally isolated in an early-pressure-based screen for recessive maternal-effect mutations [14], [17], while the *hec^p06ucal^* and *hec^p08ajug^* alleles were found in a four-generation scheme based on natural crosses ([16], [18], see also [92]). The *hec^t2800^* allele was induced in an AB/Tübingen hybrid background [14], [17], which was further hybridized with the WIK line during linkage mapping. The *hec^p08ajug^* and *hec^p06ucal^* alleles were induced in a Tübingen background, which was hybridized to an AB line in an F4 genetic screen coordinated with linkage mapping [16], [18]. Homozygous mutant *hec* fish were identified by genotyping the flanking SSLP markers z59658 and z24511, which are 1.2 cM apart on linkage group 8 and both of which were polymorphic for all three alleles. Mutant embryos were obtained by crossing homozygous *hec* females to AB males. Embryos from females homozygous mutant for the *hec^p06ucal^* allele were used unless otherwise specified. Wild-type control embryos were derived from either the AB line or heterozygous sibling females. Clutches were synchronized through 5-minute collections during natural spawning. Oocytes were collected from wild-type, *bucky ball^p106re^* [39], [43] and *magellan^p6cv^* [40] mutants. Embryos were collected and developed in E3 embryonic medium [14] and were staged according to the age and morphological standards described in [93].

For complementation tests of *hec^t2800^*, *hec^p08ajug^* and *hec^p06ucal^* mutants, homozygous mutant males of one allele were crossed with heterozygous females of another allele to produce offspring, which were raised to adulthood. Female adult fish were crossed with wild-type males and phenotyped as wild-type or ventralized mutant by examining the resulting clutches at 24 hpf. Only those clutches producing more than 50 embryos were scored and non-complementation was indicated by the presence of ventralization phenotypes similar in expressivity and penetrance to those in clutches from mutant females from the original three mutant alleles on their own.

### Isolation and genotyping of genomic DNA

Fish were anesthetized with MESAB (0.014%) and the tail fin was clipped using a razor blade and placed into 100 ul DNA lysis buffer (10 mM Tris, pH 8.0; 10 mM EDTA, pH 8.0; 200 mM NaCl; 1% Triton X-100) containing 5 µg 10 mg/ml Proteinase K. Tissue lysates were incubated overnight at 55°C, and were incubated at 94°C for 10 minutes to inactivate Proteinase K. Lysates were diluted 1∶6 with water, and 2.5 µl of this genomic DNA diluted lysate was used per 10 µl PCR reaction. For a 10 µl PCR reaction, 2 µl of GoTaq green Buffer, 0.2 µl of dNTPs, 0.05 µl of GoTaq DNA polymerase (Promega), 2.5 ul genomic DNA diluted lysate, and 1 µl each of 10 µM forward and reverse primers were used. For SSLP markers, PCR products were analyzed on a 2% high-resolution agarose gel right after the PCR reaction. For RFLP markers, PCR products were used for FastDigest Restriction Enzyme (Fermentas) digestion for 30 min, and then analyzed on a 1.5% regular agarose gel.

### Positional cloning and sequence analysis

Initial linkage was identified by bulk segregant analysis with SSLP markers. Once initial linkage of the mutation was obtained, genotypically identified homozygous mutant males were crossed to heterozygous females to generate large numbers of fish for fine mapping [92].

Chromosome walking was conducted by screening the CHORI-211 BAC library using marker z67047 and zC150E8z (0 recombination/1762 genomes). PCR-based screening of the primary pool and secondary pools identified 2 positive BAC clones. Individual BAC clones were ordered from BACPAC resources center (http://bacpac.chori.org).

To find the mutation in *hec/grip2a*, 5 fragments of *hec/grip2a* cDNA from mutant and wild-type 1-cell embryo cDNA were amplified by RT-PCR with 5 primer pairs, which cover the entire *hec/grip2a* coding region:

1. 5′-ATGTCCTGCATCTTGCTTCCAGAG-3′ and 5′-CCTCAGTGGGAATCCCATTAATGG-3′

2. 5′-TGGAGTGTTACAAGTTGGCGACAG-3′ and 5′-TGAATGGCTTCGCTCAGAGGTTTG-3′

3. 5′-TTCATATCGGTGACCGAGTTTTGG-3′ and 5′-GACATTATTGTAGCCTCAAGCTCG-3′

4. 5′-GAGACCTGCGGTCAGTCAGAAATC-3′ and 5′-GTGCTCTGTGTTTCTCATTTGTGG-3′

5. 5′-AGGACACTTCCCAACAGTCTGCAC-3′ and 5′-ACCTGATCACTTCTAACCCAACAG-3′

All PCR products were cloned into pGEM-T easy vector and sequenced.

For Phylogenetic analysis, homologous *grip1* and *grip2* genes were found using BLAST searches on Ensembl and NCBI genome databases. A phylogenetic tree was constructed and drawn using ClustalW in the MegAlign program from Lasergen. PDZ domains were identified using CD-search in the Conserved Domain Database (CDD) in NCBI [29]. Schematic diagram of the protein domain structures for each gene were drawn using DomainDraw [94].

### RT-PCR and quantitative RT-PCR

Total RNA was isolated from whole embryos using TRIzol reagent (Invitrogen). cDNA was synthesized using random primers (Invitrogen) and AMV Reverse Transcriptase (Promega). RT-PCR reactions were performed with primer pairs derived from *hec/grip2a* and *ef1α*, using 30 cycles at an annealing temperature of 58°C (in the semi-quantitative range). Absence of genomic contamination was verified by a negative control RT reaction without the Reverse Transcriptase. The following primers were used for the amplifications:

*hec/grip2a*, 5′-GAGACCTGCGGTCAGTCAGAAATC-3′ and 5′-TATGAAGCTCTAGAGGCACTGACG-3′,

*wnt8a*, 5′-CGGAAAAATGGGTGGTCGTG-3′ and 5′-AGTCGACCAGCTTCGTTGTT-3′,

*ef1α*, 5′-ACCGGCCATCTGATCTACAA-3′ and 5′-CAATGGTGATACCACGCTCA-3′.

Quantitative (q) RT-PCR was performed on an iCycler machine (Bio-Rad) using iQ SYBR Green Supermix (Bio-Rad). The thermal profile used for amplification is: 95°C for 3 min, 40 cycles of 94°C for 30 s, 58°C for 30 s and 72°C for 30 s. The relative mRNA level was quantified and normalized to *ef1α*.

### *In situ* hybridizations and antibody labeling

*In situ* hybridizations of embryos were carried out as described previously [95]. Probes for in situ included *goosecoid* [96], *chordin* [97], *even skipped 1* [98], *vasa* [33], *dazl* [46] and *wnt8a* [7], [99]. For the *grip2a in situ* probe, a fragment of *grip2a* cDNA was cloned into pGEM-T easy vector as described in the positional cloning section. Five different probes were tested against 5 different cDNA sequences, all of which showed the same expression pattern. Subsequently, all the expression data was acquired using one of the probes. Antisense digoxygenin probe was generated by linearizing and transcribing with SacII and SP6 RNA polymerase, while sense probe control was generated by linearization with SpeI and transcription with T7 RNA polymerase. Images were acquired with a Leica-FLIII microscope and a color camera (Diagnostic Instruments Spot Insight).

Ovaries were dissected from euthanized females and fixed overnight at 4°C in 4% paraformaldehyde. Fixed ovaries were then dehydrated in MeOH.

Whole mount in situ hybridization of oocytes was performed as previously described [100]. Following staining, oocytes were embedded in JB-4 Plus Plastic resin and 7 micron sections were cut using a microtome. Stained sections were coated with Permount (Fisher) prior to addition of a coverslip.

Antibody labeling of microtubules was as previously described [36]. Prior to the labeling procedure, embryos were dissected using fine dissecting forceps to generate two halves. To image cortical microtubules, bisections were carried out along an equatorial plane, the vegetal halves were labeled and mounted in 50% glycerol with DABCO reagent to prevent bleaching, with the vegetal cortex facing the coverslip. For imaging of mediolateral microtubules, bisections were carried out along a meridional plane and each mediolateral halves were labeled and mounted as above with the mediolateral cortex facing the coverslip. Images were acquired using an upright Zeiss LSM510 confocal microscope using an oil immersion 63× objective and collected as single 1.5 µm optical sections with a pinhole diameter of about 1 Airy unit, a low scan speed (preset 6) and noise filtering through a 4-pass line mode average. The resulting images were analyzed with Fiji software.

Antibody labeling of embryos to detect Sybu protein was carried in whole mount embryos using whole embryos as described previously [6], and images were acquired using a Zeiss Axioplan2 fluorescent microscope and OpenLab software.

Nocodazole treatment was carried out through exposure by 10 mpf of dechorionated embryos to a final concentration of 4 µg/ml nocodazole in E3 (diluted from a 5 mg/ml solution in DMSO), followed by fixation at the indicated periods.

Phalloidin labeling was carried out as in [36], with the exception that dechorionated embryos were labeled whole and equatorially bisected prior to mounting with the vegetal pole facing the coverslip.

### Fluorescent beads injection

Fluorescent beads injection experiments were carried as previously described [2]. A suspension of 0.2 mm fluorescent polystyrene beads (1 ul; Polysciences) was diluted in 23 *m*l water and colored with trace amounts of phenol red (0.05%). The injection solution is microinjected into the embryos near the vegetal pole using a Phemtojet microinjector (Eppendorf). Embryos had been injected at the 2-cell stage and imaged at 2 hours later. Embryos were mounted in methyl cellulose and imaged with an upright fluorescence microscope (Zeiss, Axioplan II) and a black and white digital camera (Zeiss, Axiocam).

## Supporting Information

Figure S1 Age-dependency of the *hec* mutant phenotype. The *hecate* mutant phenotype is stronger in younger females. Also note that at both ages the strength of the alleles is *hec^p06ucal^*>*hec^t2800^*>*hec^p08ajug^*. Homozygous mutant females at 3 and 12 month after birth were crossed against wild-type males to produce clutches of mutant embryos. Phenotypes were classified at 24 hpf as in [24] and Table 1. Results were pooled from clutches from 15 different females for each allele. Number of embryos is as follows: 3-month females: WT, 1440; *hec^p06ucal^*, 1178; *hec^t2800^*, 575; *hec^p08ajug^*, 1399; 12-month females: WT, 1259; *hec^p06ucal^*, 1812; *hec^t2800^*, 655; *hec^p08ajug^*, 1516. Pearson's Chi-squared test shows statistical significant differences between 3-month and 12-month females for all alleles (p-values: *hec^p06ucal^*, 0.002477; *hec^t2800^*, <2.2e-16; *hec^p08tajug^*, <2.2e-16) but not for wild-type females (p-value 0.3421). Pair-wise comparisons between all three alleles are also significantly different using the same analysis (p-values: *hec^p06ucal^* vs. *hec^t2800^* <2.2e-16; *hec^p06ucal^* vs. *hec^p08ajug^* <2.2e-16; *hec^t2800^* vs. *hec^p08ajug^* <2.2e-16).
(TIFF)

Figure S2 Phylogenetic tree of Grip1 and Grip2 proteins among Drosophila and vertebrate species and number of PDZ domains in the predicted protein. Left: phylogenetic tree using ClustalW. Gene-ID from NCBI or Ensembl-genome databases: GRIP2 rat: NP_612544.2; GRIP2 mouse: NP_001152979.1; GRIP2 human: NP_001073892.1; Grip2 *Xenopus*: NP_001091382.1; GRIP2 chicken: ENSGALP00000010397; Grip2b zebrafish: XP_001922281.1; Grip2b medaka: ENSORLP00000012064; Grip2b fugu: ENSTRUP00000040982; Grip2a fugu: ENSTRUP00000024040; Grip2a medaka: ENSORLP00000012637; Grip2a zebrafish: NP_001116760.1; GRIP1 mouse: NP_083012.1; GRIP1 rat: ENSRNOP00000061369; GRIP1 human: ENSP00000352780; GRIP1 chicken: ENSGALP00000016069; Grip1 *Xenopus*: ENSXETP00000015955; Grip1 fugu: ENSTRUP00000012549; Grip1 medaka: ENSORLP00000021825; Grip1 zebrafish: NP_001038316.1; Grip fruitfly: NP_572285.2. Right: Diagrams of the overall structure of the respective proteins (gray) highlighting the number of PDZ domains (orange), based on Ensembl annotation.
(TIFF)

Figure S3 Expression of zebrafish *grip2a* mRNA in wild-type and *hecate* mutant embryos. A) RT-PCR analysis of *grip2a* mRNA and *ef1α* control expression in wild-type and *hec* mutant embryos, as well as wild-type ovaries and wild-type adults (male, female, female with removed ovaries). B) Quantitative RT-PCR analysis shows *grip2a* mRNA expression levels, relative to *ef1α* expression at the same stages. Maternal *grip2a* mRNA levels are reduced in embryos mutant for all *hec* alleles.
(TIFF)

Figure S4 Quantification of the off-center shift of *grip2a* mRNA localization domain in control (DMSO-treated) and nocodazole-treated embryos fixed at 40 mpf. A) Diagram indicating angles of the *grip2a* mRNA localization domain landmarks with respect to the vegetal pole of the embryo. LB: left boundary; RB: right boundary; S: total span of domain; M: midpoint of domain. B) Quantification parameters, with brackets indicating standard deviation. In control embryos, but not in nocodazole-treated embryos, left and right boundaries and midpoint of the *grip2a* mRNA localization domain experience a similar off-center shift, while the span of the domain appears unchanged. Blind analysis of 38 and 41 DMSO- and nocodazole-treated embryos, respectively. Similar changes were observed in embryos fixed at 30 mpf, although differences at this time point were less pronounced than at 40 mpf (data not shown).
(TIF)

Figure S5 Distribution of microtubule organization phenotypes at the vegetal cortex of wild-type and *hecate* mutant embryos. A–G) Examples of various vegetal cortex microtubule arrangements at 20 mpf: normally aligned (A), partially aligned (B, C), unbundled and lacking organization (D, E) and exhibiting aster-like structures (F, G). (A) is from a wild-type embryo and (B–G) from *hec* mutants. Magnification bar in (G) corresponds to 40 µm for panels (A–G). H) Distribution of phenotypes. Wild-type embryos exhibit highly aligned microtubule network while *hec* mutants show disorganization, lack of bundling and aster-like structures. The two distributions are significantly different (Fisher's Exact Test, p-value = 5.335e-09).
(TIF)

Figure S6 Treatment with taxol does not affect the *hec* mutant phenotype. Phenotypic distribution of axial defects (classification as in [24]) of taxol- and solvent (DMSO)-treated wild-type and *hec* mutant embryos. Data is derived from two to three different experiments after a 30 minute exposure to 10 µM taxol starting at 10 mpf. There are no statistically significant phenotypic differences between taxol-treated and control embryos (student t-test, 2-tailed, unpaired).
(TIFF)

Figure S7 F-actin cortex at the vegetal pole is similar in wild-type and *hec* mutant embryos. At 20 mpf, both wild-type (A) and mutant (B) eggs show F-actin rich folds and villi-like structures, which may correspond to previously described microplicae [45]. Number of embryos tested: 18 wt (from a pool of four females) and 24 mutants (from two different mutant females). A fraction (21%, n = 24) of *hec* mutant embryos show radial F-actin enrichments (C), correlating with aster-like microtubule structures in these embryos (Figure S5). Magnification bar in (C) corresponds to 40 µm in all panels.
(TIFF)

Figure S8 Expression of *wnt8a* mRNA in *hec* mutant embryos. Quantitative RT-PCR analysis of *wnt8a* mRNA levels relative to *ef1α* mRNA. *wnt8a* relative expression in *hec* mutants is close to wild-type at 30 mpf but becomes reduced at 60 mpf.
(TIFF)

Figure S9 PGC determination is not adversely affected in *hecate* mutant embryos. The number of PGCs (as identified by *vasa* expression through whole mount in situ hybridization) was determined in wild-type and *hec* mutant embryos at the 3- through 5-somite stages (ca. 10.5 hpf). At these stages, PGCs have not yet reached the prospective gonad location and appear relatively scattered, which facilitated quantification analysis. The average number of PGCs was 17.9 +/− 3.5 in *hec* mutant embryos (n embryos = 158), compared to 13.6 +/− 3.6 in wild-type embryos (n embryos = 214). The slightly higher number of PGCs in mutants was statistically significant (Student's t-test, p-value = 0.02). It is possible, however, that this difference reflects a bias in our ability to count individual PGCs in *hec* mutants, where PGCs are dispersed throughout the span of the embryos, compared to wild-type embryos, where PGCs are concentrated along the dorsal axis. Regardless of this uncertainty, the data indicate that *hec* function is not essential for PGCs determination.
(TIFF)

Table S1 Three identified *hec* mutant alleles do not complement each other in complementation crosses. Top: Homozygous mutant males of one allele were crossed with heterozygous females of another allele to produce offsprings, which are raised to adulthood. Female adult F_1_ progeny were tested for *hec*-associated maternal effects by crossing them against wild-type males and scoring the resulting F_2_ clutches as wild-type or mutant. Only test crosses with more than 50 embryos were scored, and were scored as mutant when the fraction of V1–V4 categories was ≥50%. F2 clutches scored as wild-type exhibited ≤2% of embryos with V1–V4 phenotypes. If mutations are allelic, crosses are expected to yield F1 females exhibiting wild-type (from heterozygote females) or mutant (from transheterozygote females) F_2_ phenotypes in a 1∶1 ratio. Bottom: Summary of analysis showing non-complementation of three *hec* alleles.
(DOC)
