# Correction: Hecate/Grip2a Acts to Reorganize the Cytoskeleton in the Symmetry-Breaking Event of Embryonic Axis Induction

**DOI:** 10.1371/journal.pgen.1004786

**Published:** 2014-10-15

**Authors:** The PLOS Genetics Staff

The stated Author Contributions for Elliot W. Abrams and Tripti Gupta are incomplete. In addition to contributing reagents/materials/analysis tools, both of these authors also conceived and designed experiments, performed experiments and analyzed data. The correct author contributions for this manuscript are as follows:

Conceived and designed the experiments: XG DG EW MCM FP EWA TG. Performed the experiments: XG DG EW CH JLG ED TY AA EWA TG. Analyzed the data: XG DG EW JLG TY EWA TG. Contributed reagents/materials/analysis tools: EWA TG FLM MCM. Wrote the paper: XG EW MCM FP.
