# Supplementary figures and images for: Hecate/Grip2a Acts to Reorganize the Cytoskeleton in the Symmetry-Breaking Event of Embryonic Axis Induction
Source: PLoS Genet. 2014 Jun 26;10(6):e1004422. doi: 10.1371/journal.pgen.1004422 (PMC4072529; doi:10.1371/journal.pgen.1004422)

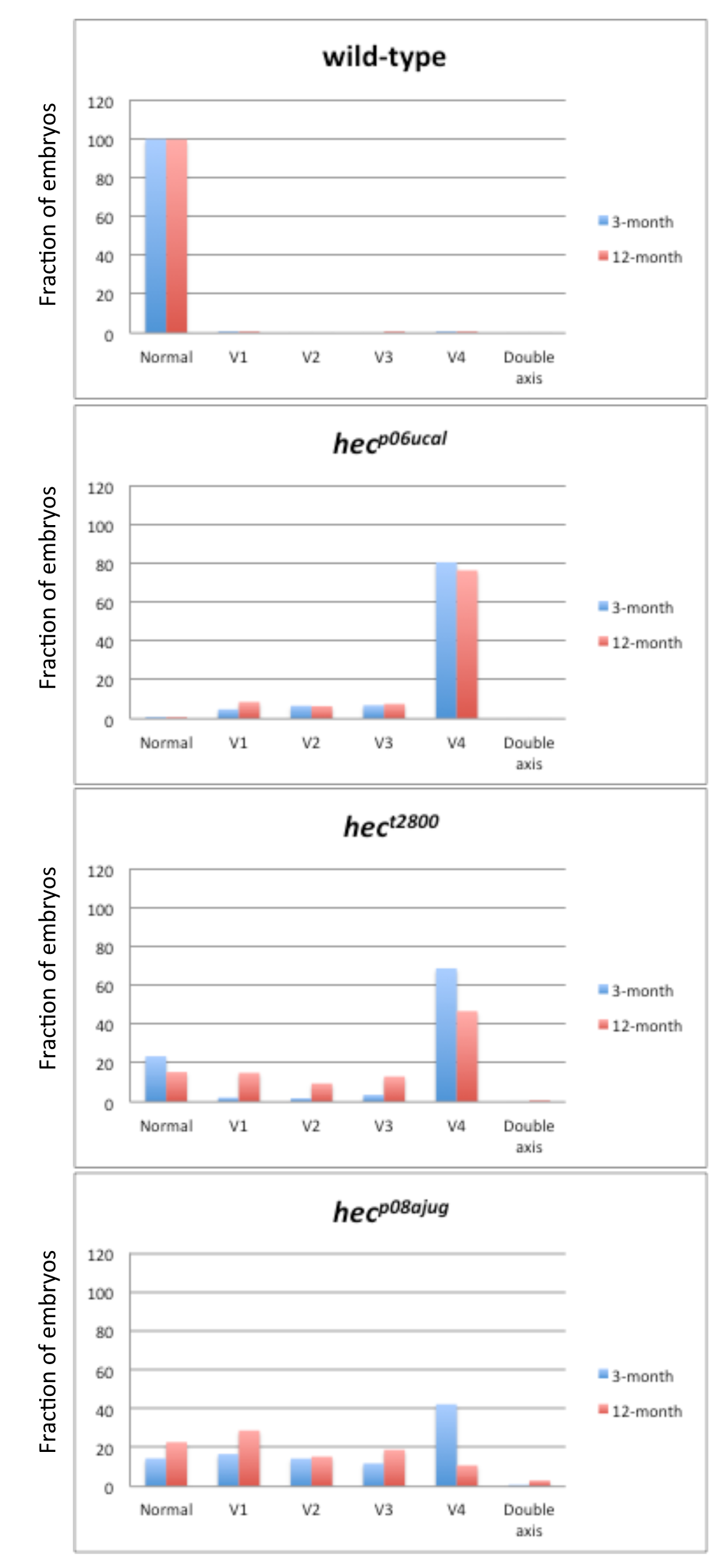

Supplement: Figure S1 — Age-dependency of the hec mutant phenotype. The hecate mutant phenotype is stronger in younger females. Also note that at both ages the strength of the alleles is hecp06ucal>hect2800>hecp08ajug. Homozygous mutant females at 3 and 12 month after birth were crossed against wild-type males to produce clutches of mutant embryos. Phenotypes were classified at 24 hpf as in [24] and Table 1. Results were pooled from clutches from 15 different females for each allele. Number of embryos is as follows: 3-month females: WT, 1440; hecp06ucal, 1178; hect2800, 575; hecp08ajug, 1399; 12-month females: WT, 1259; hecp06ucal, 1812; hect2800, 655; hecp08ajug, 1516. Pearson's Chi-squared test shows statistical significant differences between 3-month and 12-month females for all alleles (p-values: hecp06ucal, 0.002477; hect2800, <2.2e-16; hecp08tajug, <2.2e-16) but not for wild-type females (p-value 0.3421). Pair-wise comparisons between all three alleles are also significantly different using the same analysis (p-values: hecp06ucal vs. hect2800 <2.2e-16; hecp06ucal vs. hecp08ajug <2.2e-16; hect2800 vs. hecp08ajug <2.2e-16). (TIFF) [file pgen.1004422.s001.tiff]

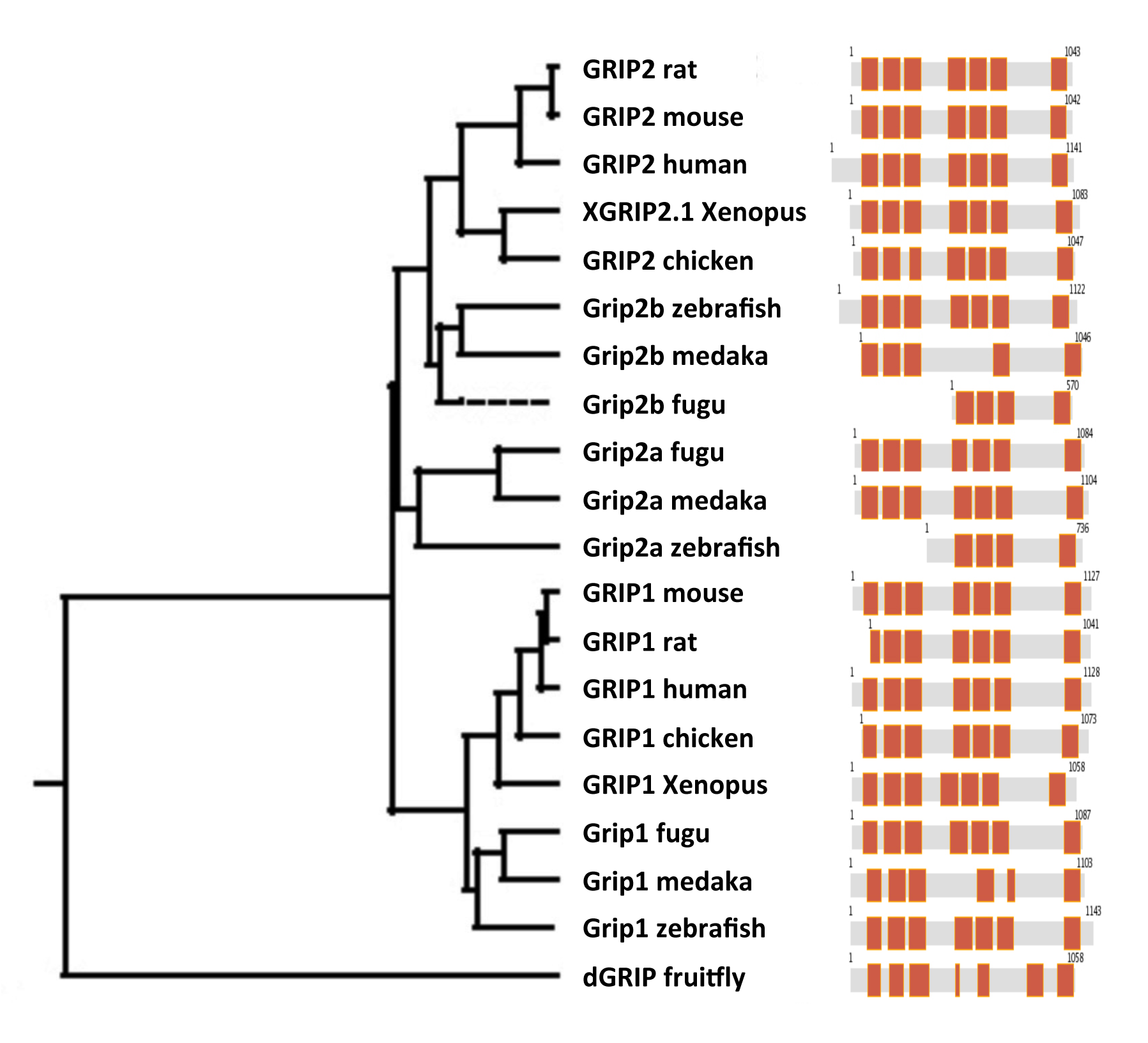

Supplement: Figure S2 — Phylogenetic tree of Grip1 and Grip2 proteins among Drosophila and vertebrate species and number of PDZ domains in the predicted protein. Left: phylogenetic tree using ClustalW. Gene-ID from NCBI or Ensembl-genome databases: GRIP2 rat: NP_612544.2; GRIP2 mouse: NP_001152979.1; GRIP2 human: NP_001073892.1; Grip2 Xenopus: NP_001091382.1; GRIP2 chicken: ENSGALP00000010397; Grip2b zebrafish: XP_001922281.1; Grip2b medaka: ENSORLP00000012064; Grip2b fugu: ENSTRUP00000040982; Grip2a fugu: ENSTRUP00000024040; Grip2a medaka: ENSORLP00000012637; Grip2a zebrafish: NP_001116760.1; GRIP1 mouse: NP_083012.1; GRIP1 rat: ENSRNOP00000061369; GRIP1 human: ENSP00000352780; GRIP1 chicken: ENSGALP00000016069; Grip1 Xenopus: ENSXETP00000015955; Grip1 fugu: ENSTRUP00000012549; Grip1 medaka: ENSORLP00000021825; Grip1 zebrafish: NP_001038316.1; Grip fruitfly: NP_572285.2. Right: Diagrams of the overall structure of the respective proteins (gray) highlighting the number of PDZ domains (orange), based on Ensembl annotation. (TIFF) [file pgen.1004422.s002.tiff]

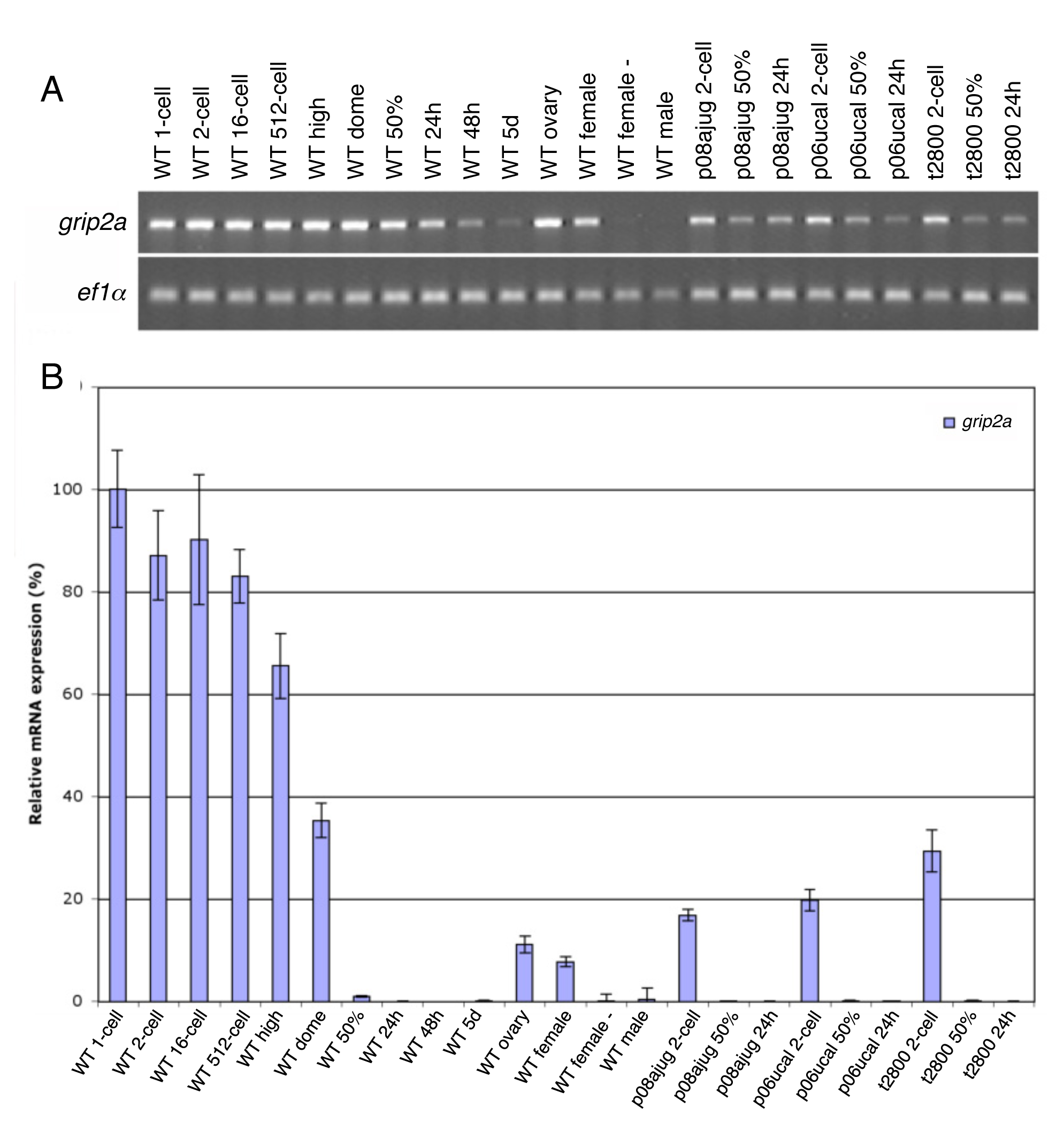

Supplement: Figure S3 — Expression of zebrafish grip2a mRNA in wild-type and hecate mutant embryos. A) RT-PCR analysis of grip2a mRNA and ef1α control expression in wild-type and hec mutant embryos, as well as wild-type ovaries and wild-type adults (male, female, female with removed ovaries). B) Quantitative RT-PCR analysis shows grip2a mRNA expression levels, relative to ef1α expression at the same stages. Maternal grip2a mRNA levels are reduced in embryos mutant for all hec alleles. (TIFF) [file pgen.1004422.s003.tiff]

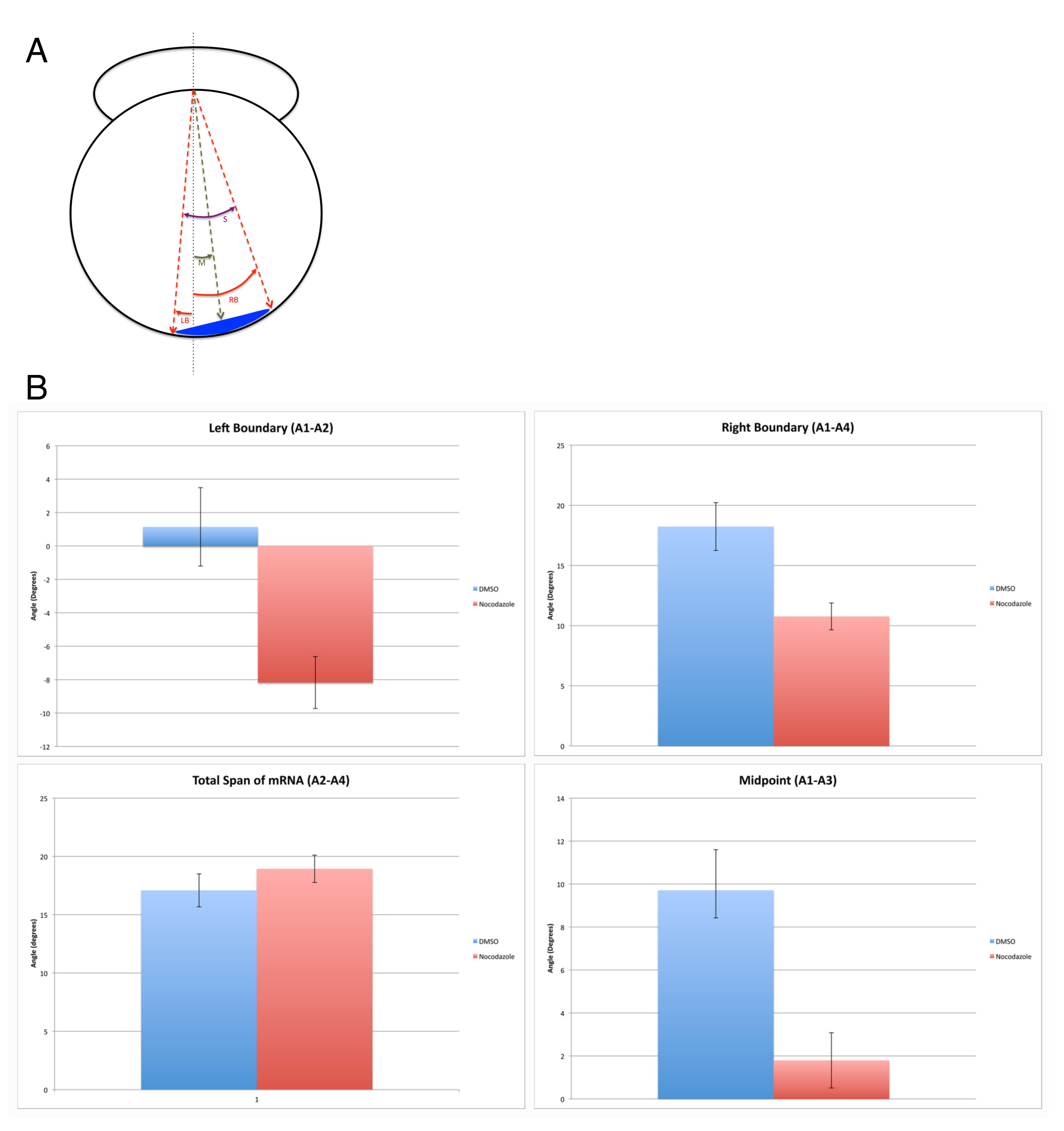

Supplement: Figure S4 — Quantification of the off-center shift of grip2a mRNA localization domain in control (DMSO-treated) and nocodazole-treated embryos fixed at 40 mpf. A) Diagram indicating angles of the grip2a mRNA localization domain landmarks with respect to the vegetal pole of the embryo. LB: left boundary; RB: right boundary; S: total span of domain; M: midpoint of domain. B) Quantification parameters, with brackets indicating standard deviation. In control embryos, but not in nocodazole-treated embryos, left and right boundaries and midpoint of the grip2a mRNA localization domain experience a similar off-center shift, while the span of the domain appears unchanged. Blind analysis of 38 and 41 DMSO- and nocodazole-treated embryos, respectively. Similar changes were observed in embryos fixed at 30 mpf, although differences at this time point were less pronounced than at 40 mpf (data not shown). (TIF) [file pgen.1004422.s004.tif]

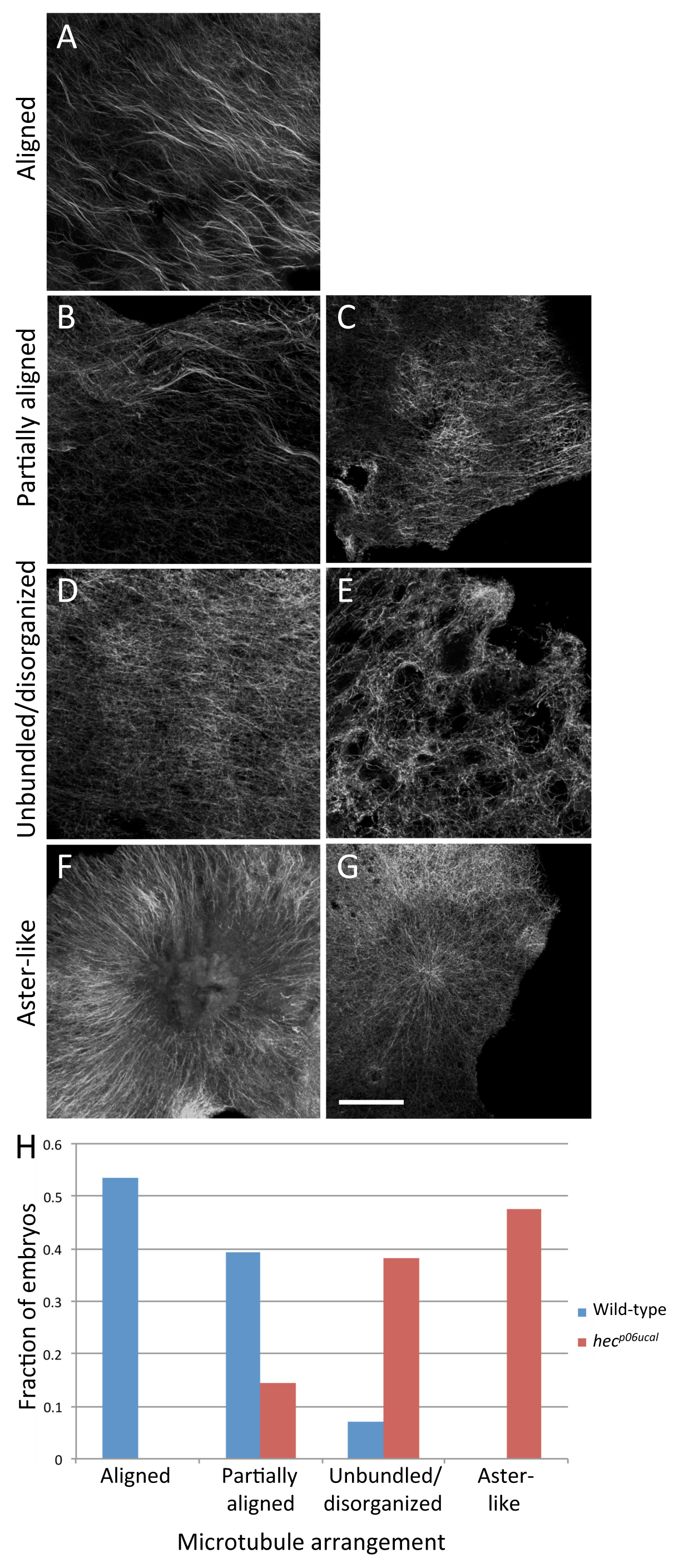

Supplement: Figure S5 — Distribution of microtubule organization phenotypes at the vegetal cortex of wild-type and hecate mutant embryos. A–G) Examples of various vegetal cortex microtubule arrangements at 20 mpf: normally aligned (A), partially aligned (B, C), unbundled and lacking organization (D, E) and exhibiting aster-like structures (F, G). (A) is from a wild-type embryo and (B–G) from hec mutants. Magnification bar in (G) corresponds to 40 µm for panels (A–G). H) Distribution of phenotypes. Wild-type embryos exhibit highly aligned microtubule network while hec mutants show disorganization, lack of bundling and aster-like structures. The two distributions are significantly different (Fisher's Exact Test, p-value = 5.335e-09). (TIF) [file pgen.1004422.s005.tif]

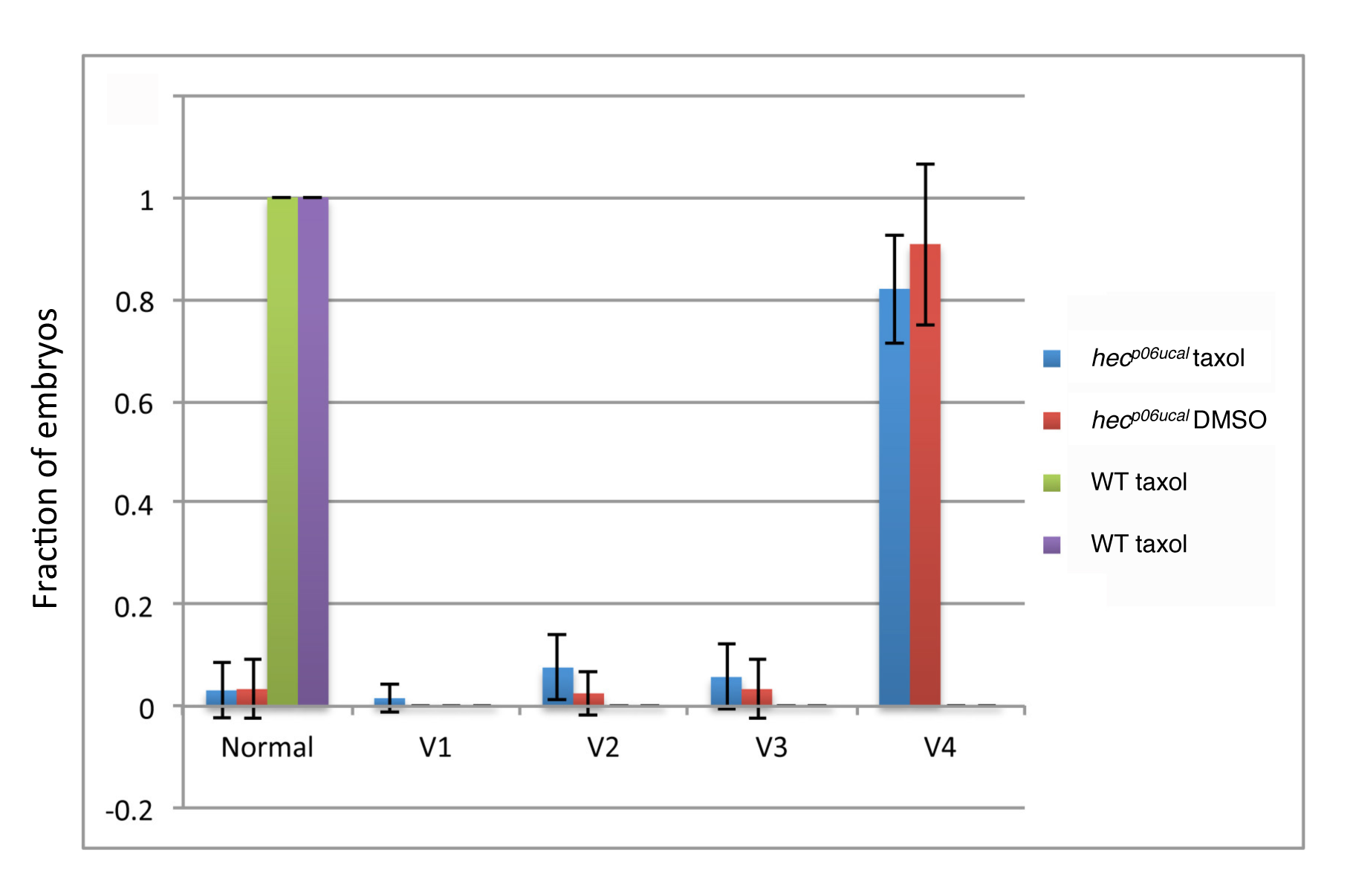

Supplement: Figure S6 — Treatment with taxol does not affect the hec mutant phenotype. Phenotypic distribution of axial defects (classification as in [24]) of taxol- and solvent (DMSO)-treated wild-type and hec mutant embryos. Data is derived from two to three different experiments after a 30 minute exposure to 10 µM taxol starting at 10 mpf. There are no statistically significant phenotypic differences between taxol-treated and control embryos (student t-test, 2-tailed, unpaired). (TIFF) [file pgen.1004422.s006.tiff]

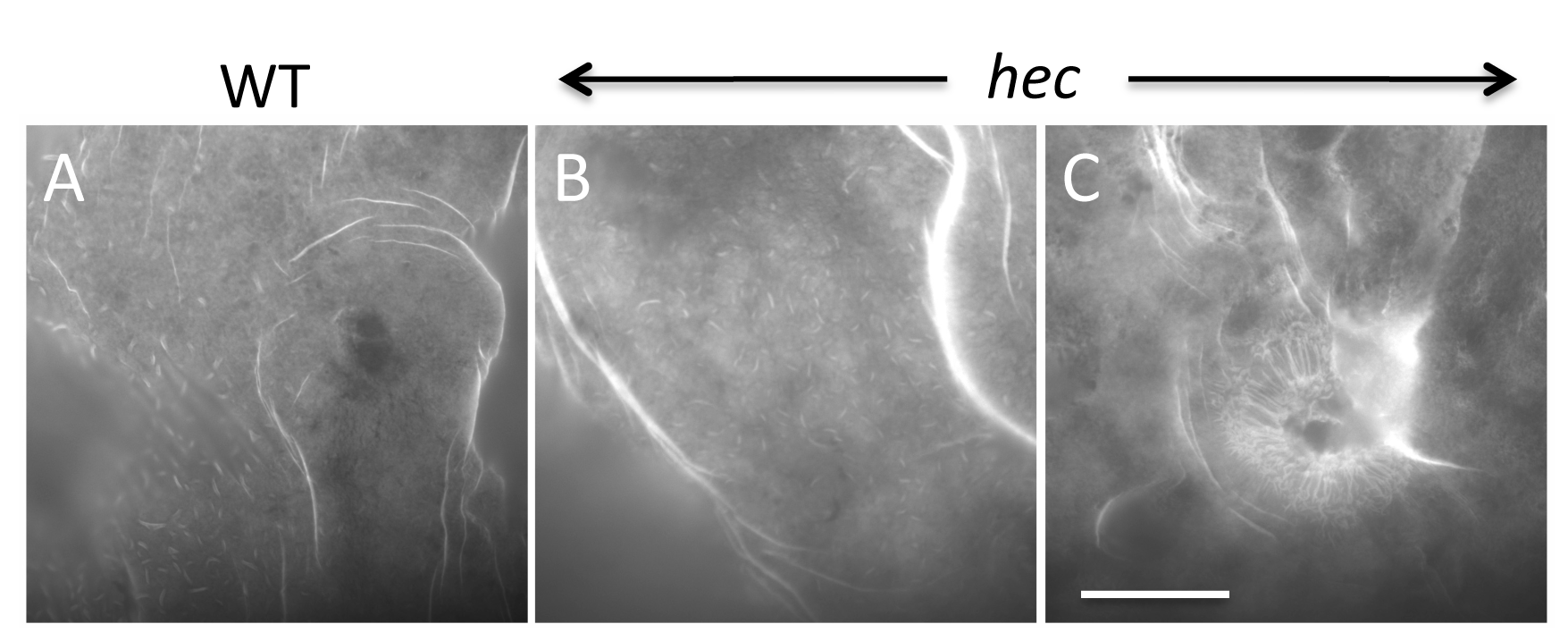

Supplement: Figure S7 — F-actin cortex at the vegetal pole is similar in wild-type and hec mutant embryos. At 20 mpf, both wild-type (A) and mutant (B) eggs show F-actin rich folds and villi-like structures, which may correspond to previously described microplicae [45]. Number of embryos tested: 18 wt (from a pool of four females) and 24 mutants (from two different mutant females). A fraction (21%, n = 24) of hec mutant embryos show radial F-actin enrichments (C), correlating with aster-like microtubule structures in these embryos (Figure S5). Magnification bar in (C) corresponds to 40 µm in all panels. (TIFF) [file pgen.1004422.s007.tiff]

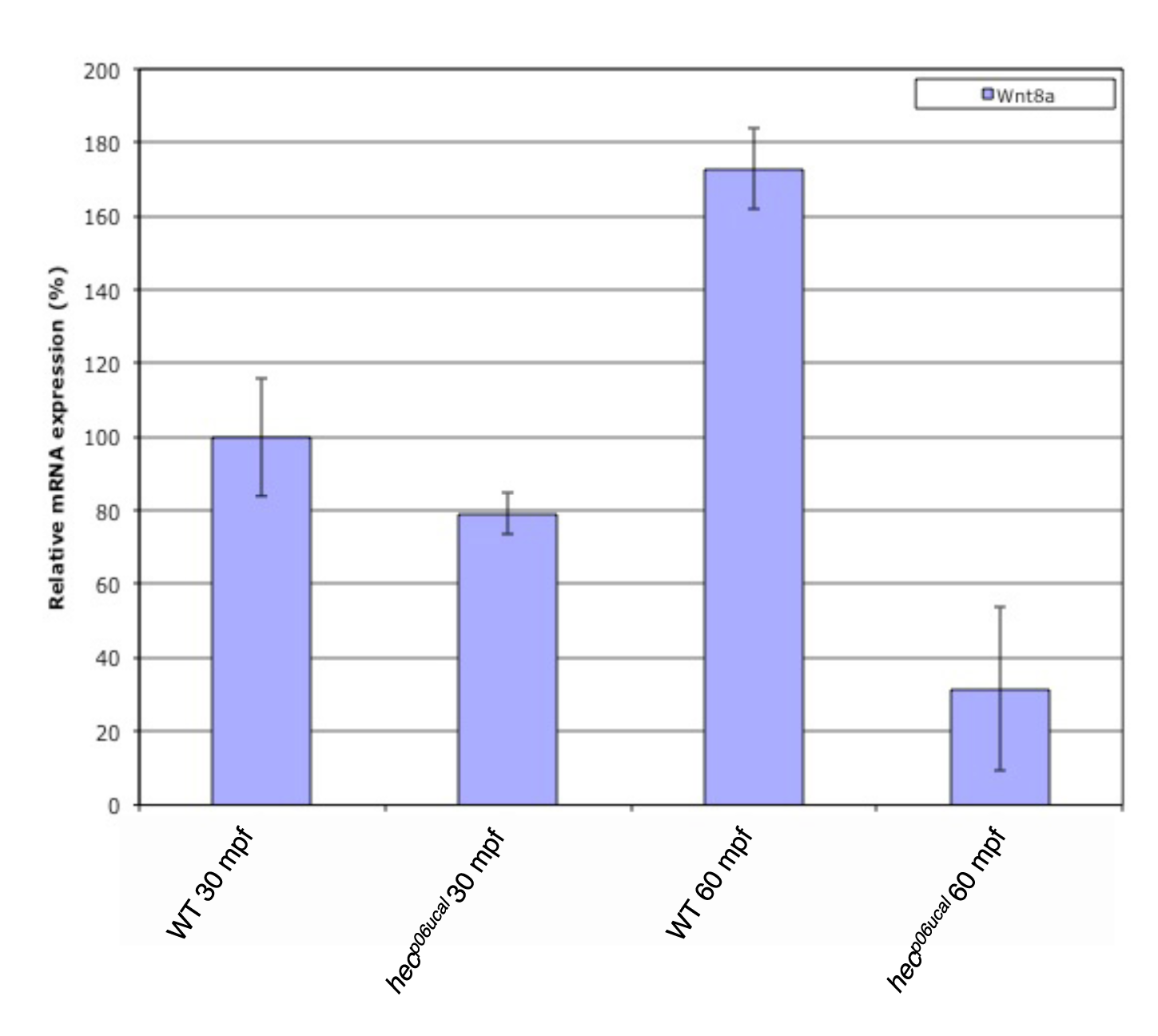

Supplement: Figure S8 — Expression of wnt8a mRNA in hec mutant embryos. Quantitative RT-PCR analysis of wnt8a mRNA levels relative to ef1α mRNA. wnt8a relative expression in hec mutants is close to wild-type at 30 mpf but becomes reduced at 60 mpf. (TIFF) [file pgen.1004422.s008.tiff]

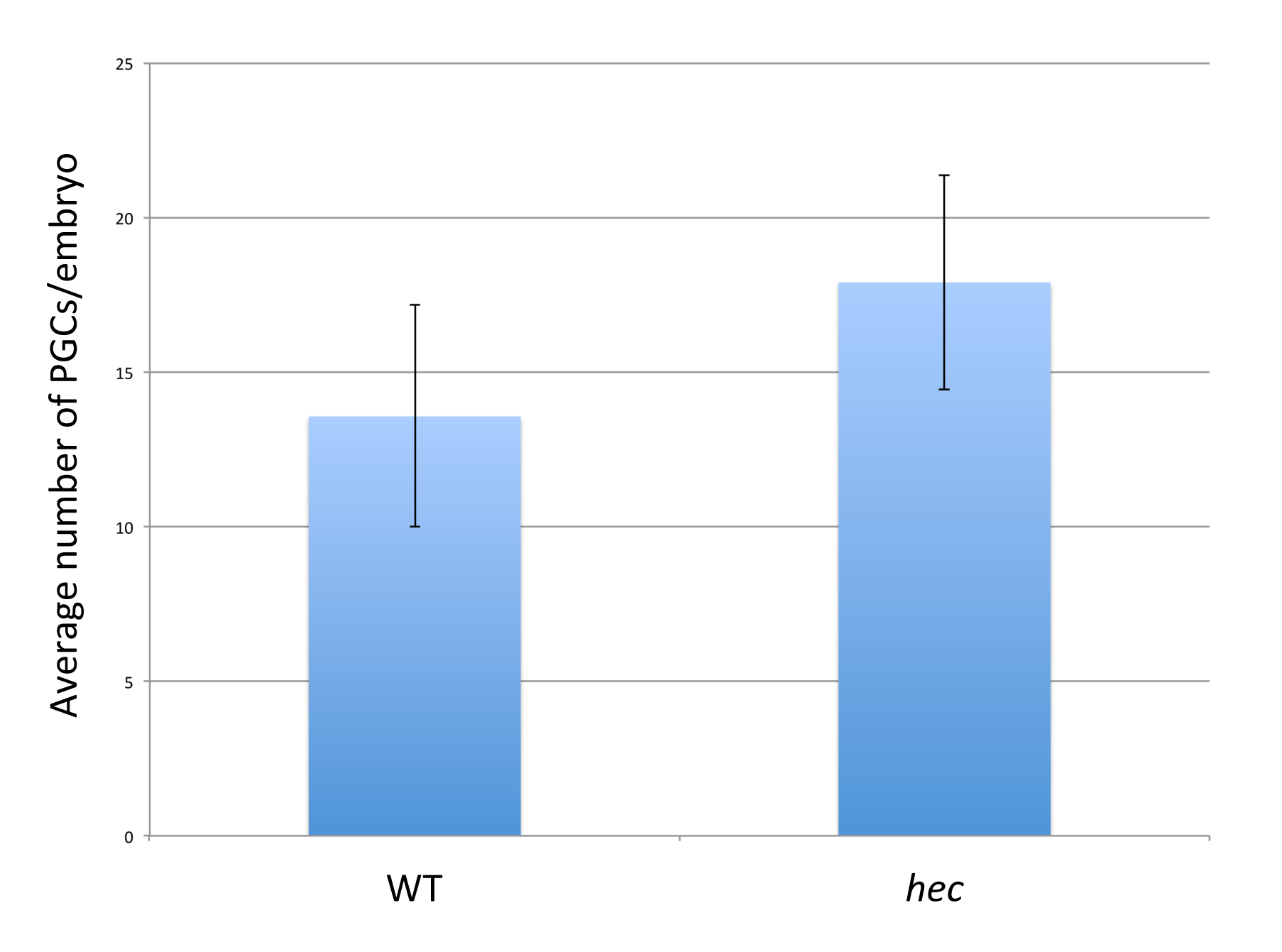

Supplement: Figure S9 — PGC determination is not adversely affected in hecate mutant embryos. The number of PGCs (as identified by vasa expression through whole mount in situ hybridization) was determined in wild-type and hec mutant embryos at the 3- through 5-somite stages (ca. 10.5 hpf). At these stages, PGCs have not yet reached the prospective gonad location and appear relatively scattered, which facilitated quantification analysis. The average number of PGCs was 17.9 +/− 3.5 in hec mutant embryos (n embryos = 158), compared to 13.6 +/− 3.6 in wild-type embryos (n embryos = 214). The slightly higher number of PGCs in mutants was statistically significant (Student's t-test, p-value = 0.02). It is possible, however, that this difference reflects a bias in our ability to count individual PGCs in hec mutants, where PGCs are dispersed throughout the span of the embryos, compared to wild-type embryos, where PGCs are concentrated along the dorsal axis. Regardless of this uncertainty, the data indicate that hec function is not essential for PGCs determination. (TIFF) [file pgen.1004422.s009.tiff]
