# Supplementary material for: Hecate/Grip2a Acts to Reorganize the Cytoskeleton in the Symmetry-Breaking Event of Embryonic Axis Induction
Source: PLoS Genet. 2014 Jun 26;10(6):e1004422. doi: 10.1371/journal.pgen.1004422 (PMC4072529; doi:10.1371/journal.pgen.1004422)
Supplement: Table S1 — Three identified hec mutant alleles do not complement each other in complementation crosses. Top: Homozygous mutant males of one allele were crossed with heterozygous females of another allele to produce offsprings, which are raised to adulthood. Female adult F1 progeny were tested for hec-associated maternal effects by crossing them against wild-type males and scoring the resulting F2 clutches as wild-type or mutant. Only test crosses with more than 50 embryos were scored, and were scored as mutant when the fraction of V1–V4 categories was ≥50%. F2 clutches scored as wild-type exhibited ≤2% of embryos with V1–V4 phenotypes. If mutations are allelic, crosses are expected to yield F1 females exhibiting wild-type (from heterozygote females) or mutant (from transheterozygote females) F2 phenotypes in a 1∶1 ratio. Bottom: Summary of analysis showing non-complementation of three hec alleles. (DOC) [file pgen.1004422.s010.doc]

**Table S1: Complementation analysis of *hec^t2800^*, *hec^p06ucal^* and *hec^p08ajug^* alleles.**

|  | wild-type | mutant |
| --- | --- | --- |
| *p06ucal* / *p06ucal* * *p08ajug* / + | 4 | 8 |
| *p06ucal* / *p06ucal* * *t2800* / + | 11 | 10 |
| *t2800* / *t2800* * *p08ajug* / + | 8 | 11 |
| *t2800* / *t2800* * *p06ucal* / + | 1 | 4 |
| *p08ajug* / *p08ajug* * *t2800* / + | 8 | 11 |
| *p08ajug* / *p08ajug* * *p06ucal* / + | 22 | 14 |

|  | *hecate^p06ucal^* | *hecate^t2800^* | *hecate^p08ajug^* |
| --- | --- | --- | --- |
| *hecate^p06ucal^* | mutant | mutant | mutant |
| *hecate^t2800^* |  | mutant | mutant |
| *hecate^p08ajug^* |  |  | mutant |
